# Cardiac Plin5 interacts with SERCA2 and promotes calcium handling and cardiomyocyte contractility

**DOI:** 10.26508/lsa.202201690

**Published:** 2023-01-30

**Authors:** Mathieu Cinato, Ismena Mardani, Azra Miljanovic, Christina Drevinge, Marion Laudette, Entela Bollano, Marcus Henricsson, Johan Tolö, Marcos Bauza Thorbrügge, Max Levin, Malin Lindbom, Muhammad Arif, Pal Pacher, Linda Andersson, Charlotta S Olofsson, Jan Borén, Malin C Levin

**Affiliations:** 1 Department of Molecular and Clinical Medicine/Wallenberg Laboratory, Institute of Medicine, Sahlgrenska Academy at University of Gothenburg and Sahlgrenska University Hospital, Gothenburg, Sweden; 2 Department of Physiology/Metabolic Physiology, Institute of Neuroscience and Physiology, Sahlgrenska Academy at University of Gothenburg , Gothenburg, Sweden; 3 Laboratory of Cardiovascular Physiology and Tissue Injury, National Institute on Alcohol Abuse and Alcoholism, National Institutes of Health, Bethesda, MD, USA

## Abstract

The adult heart develops hypertrophy to reduce ventricular wall stress and maintain cardiac function in response to an increased workload. Although pathological hypertrophy generally progresses to heart failure, physiological hypertrophy may be cardioprotective. Cardiac-specific overexpression of the lipid-droplet protein perilipin 5 (Plin5) promotes cardiac hypertrophy, but it is unclear whether this response is beneficial. We analyzed RNA-sequencing data from human left ventricle and showed that cardiac *PLIN5* expression correlates with up-regulation of cardiac contraction–related processes. To investigate how elevated cardiac Plin5 levels affect cardiac contractility, we generated mice with cardiac-specific overexpression of *Plin5* (MHC-*Plin5* mice). These mice displayed increased left ventricular mass and cardiomyocyte size but preserved heart function. Quantitative proteomics identified sarcoplasmic/endoplasmic reticulum Ca^2+^ ATPase 2 (SERCA2) as a Plin5-interacting protein. In situ proximity ligation assay further confirmed the Plin5/SERCA2 interaction. Live imaging showed increases in intracellular Ca^2+^ release during contraction, Ca^2+^ removal during relaxation, and SERCA2 function in MHC-*Plin5* versus WT cardiomyocytes. These results identify a role of Plin5 in improving cardiac contractility through enhanced Ca^2+^ signaling.

## Abstract (precis)

We show that elevated cardiac Plin5 correlates with up-regulation of cardiac contraction–related processes, unraveling a novel Plin5 interaction with SERCA2 associated with improved calcium handling.

## Introduction

Cardiac hypertrophy is generally considered a poor prognostic sign, often progressing to severe heart failure (Braunwald, 2013). However, cardiac hypertrophy can be induced by physiological stimuli such as exercise (Vega et al, 2017; Nakamura & Sadoshima, 2018). Physiological hypertrophic growth of the heart is characterized by improved cardiovascular performance, and enhanced metabolic efficiency and oxidative capacity (Nakamura & Sadoshima, 2018), and emerging evidence suggests that such physiological remodeling may actually be cardioprotective (Schüttler et al, 2019). Understanding pathways that are associated with physiological hypertrophy could thus potentially identify new therapeutic targets for the prevention and treatment of cardiovascular disease.

Perilipin 5 (Plin5) is a lipid-droplet protein that is highly expressed in the heart (Dalen et al, 2007). In addition to mediating lipid-droplet function, Plin5 is reported to be a transcriptional co-regulator, regulating processes such as mitochondrial biogenesis and oxidative capacity (Kimmel & Sztalryd, 2014; Gallardo-Montejano et al, 2016; Andersson et al, 2017). Earlier studies have shown that cardiomyocyte-specific overexpression of *Plin5* promotes cardiac hypertrophy without compromising heart function (Wang et al, 2013; Kolleritsch et al, 2019), suggesting that Plin5 may be involved in mediating physiological hypertrophy. We have previously shown that mice deficient in *Plin5* have reduced cardiac metabolic flexibility and impaired heart function after cardiac stress, such as myocardial infarction (Drevinge et al, 2016). We have also shown that reduced levels of cardiac *Plin5* correlate with reduced heart function in humans (Drevinge et al, 2016). Together, these observations suggest that elevating cardiac *Plin5* may be protective.

In this study, we analyzed human RNA-sequencing (RNA-seq) data from the left ventricle and showed that high *PLIN5* expression in the heart correlates with up-regulation of genes related to cardiac contraction. To determine how elevated cardiac Plin5 levels affect cardiac contractility, we generated mice with cardiac-specific overexpression of *Plin5* (MHC-*Plin5* mice) and identified a role of Plin5 in Ca^2+^ signaling.

## Results and Discussion

### *PLIN5* expression in the human left ventricle correlates with cardiac contractility–related processes

**Figure 1.**
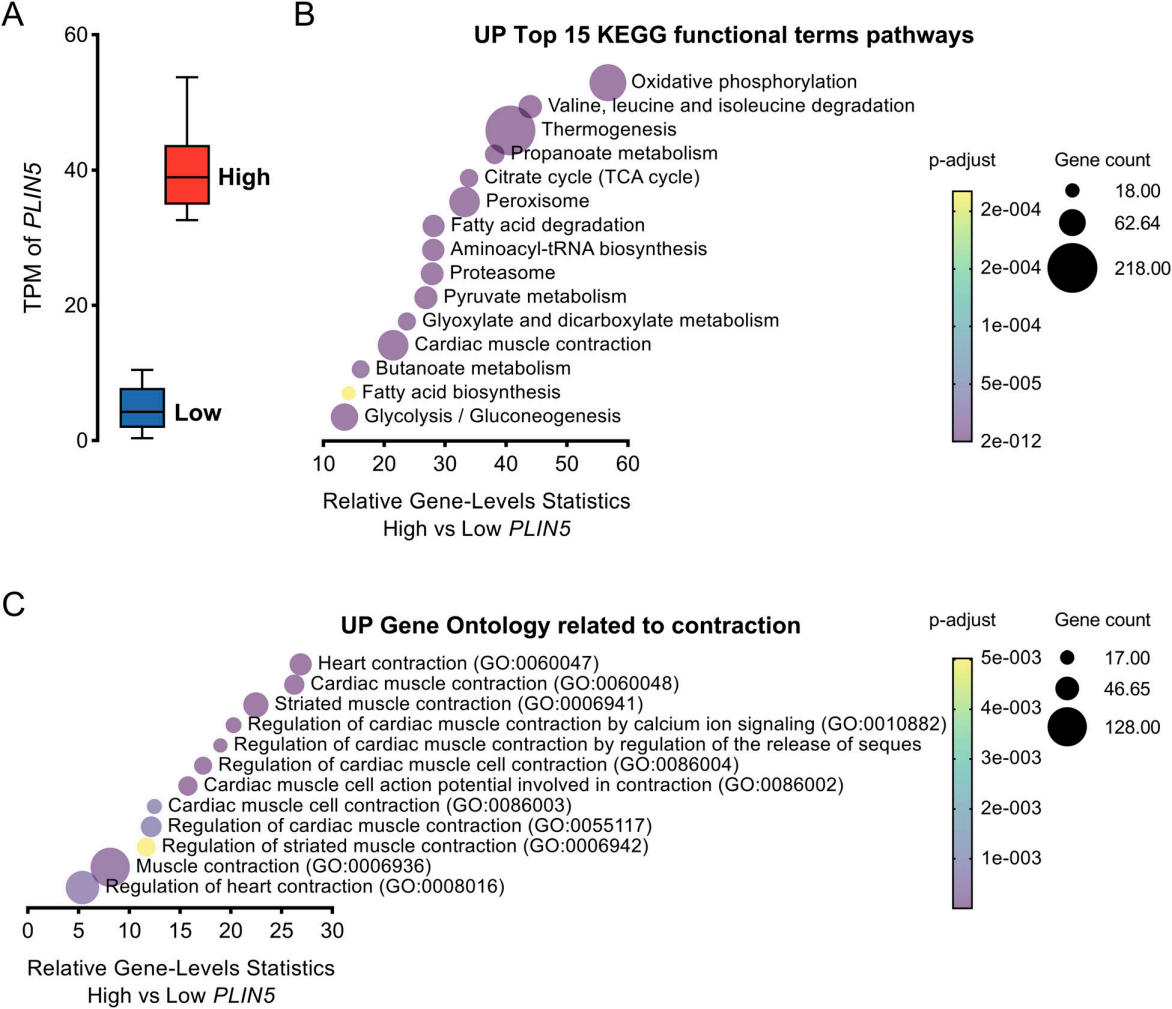
High *Plin5* expression in human heart is associated with up-regulation of cardiac contraction. **(A)** Transcript per kilobase million of *PLIN5* in the left ventricle from donors with values in the bottom and top quartiles, indicating low and high *PLIN5* expression, respectively (106 samples per group). **(B)** Top 15 up-regulated functional terms from the KEGG pathway enrichment analysis of genes that were differentially expressed in heart tissues from humans with high versus low *PLIN5* expression. **(C)** Up-regulated gene ontology terms related to contraction from the analysis of genes that were differentially expressed in heart tissues from humans with high versus low *PLIN5* expression. Source data are available for this figure.

To investigate whether high expression levels of *PLIN5* in the human heart associate with heart function, we analyzed human RNA-seq data generated by the Genotype-Tissue Expression project. We extracted data on *PLIN5* in the left ventricle from 432 donors and assigned those with *PLIN5* transcript per kilobase million (TPM) values in the top and bottom quantiles to high and low *PLIN5* expression groups, respectively. The *PLIN5* TPM value in the high-expression group was about eight times higher than in the low-expression group (Fig 1A). We conducted Kyoto Encyclopedia of Genes and Genomes (KEGG) pathway enrichment analyses of genes that were differentially expressed between the high and low *PLIN5* expression groups. The top 15 KEGG functional terms that were up-regulated in the high *PLIN5* expression group included not only known functions of Plin5, such as oxidative phosphorylation, thermogenesis, and fatty acid degradation (Mason & Watt, 2015; Najt et al, 2020; Gallardo-Montejano et al, 2021), but also cardiac muscle contraction (Fig 1B). Our enrichment analysis also identified up-regulated gene ontology terms related to contraction in the group with high versus low *PLIN5* expression (Fig 1C). These data thus support the hypothesis that elevating cardiac Plin5 levels is protective. However, we only show correlations and cannot infer causality between Plin5 levels and cardiac contractility using this material.

### MHC-*Plin5* mice develop spontaneous physiological cardiac hypertrophy

**Figure S1.**
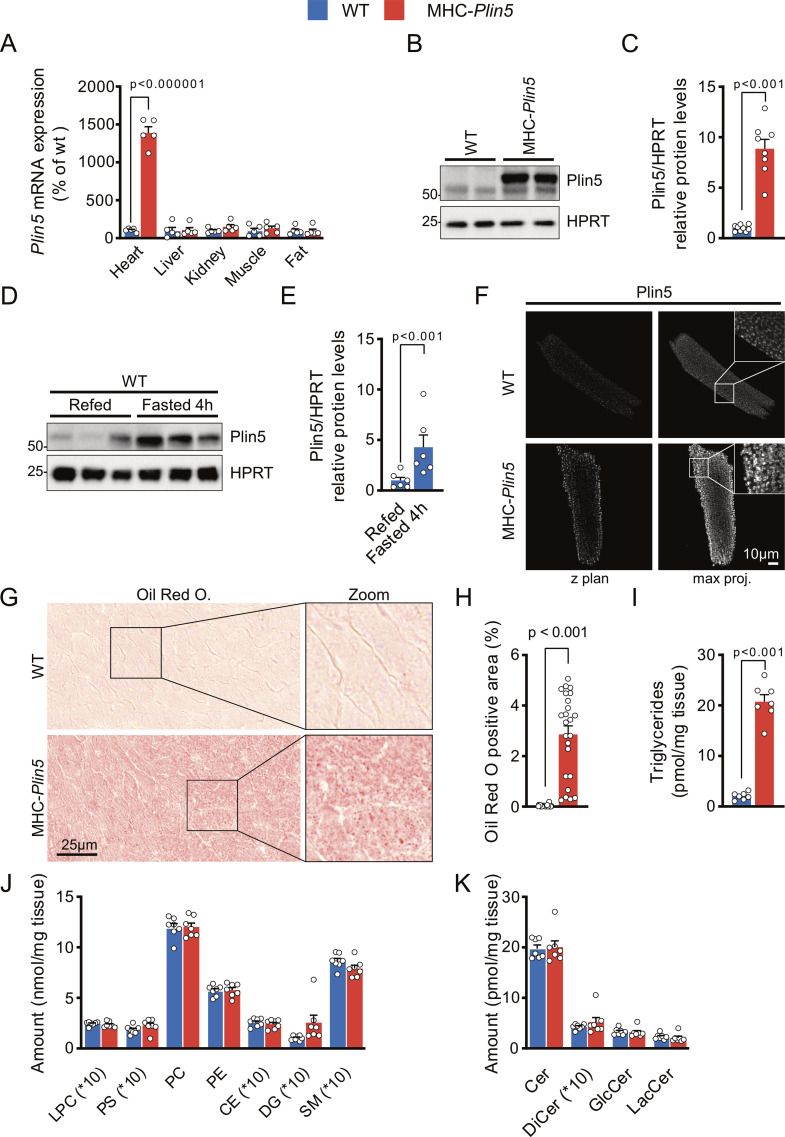
Characterization of MHC-*Plin5* mice. **(A)** Heart, liver, kidney, fat, and skeletal muscle from WT and MHC-*Plin5* mice were analyzed for relative mRNA levels of Plin5 (n_heart_ = 5 [WT], 5 [MHC-*Plin5*]). **(B)** Representative immunoblot images of Plin5 in hearts from WT and MHC-*Plin5* mice. HPRT served as a loading control. **(C)** Quantification of (B) (n_heart_ = 9 [WT], 8 [MHC-*Plin5*]). **(D)** Representative immunoblot images of Plin5 in hearts from refed and 4-h fasted WT mice. HPRT served as a loading control. **(E)** Quantification of (D) (n_mice_ = 6 [WT], 6 [MHC-*Plin5*]). **(F)** Representative single z-plane confocal scan and respective 2D maximum-intensity projection along the z-axis of immunofluorescent staining of Plin5 in a WT and an MHC-*Plin5* cardiomyocyte. Scale bar, 10 *µ*m. **(G)** Representative heart cryosections of WT and MHC-*Plin5* mice stained with Oil Red O. Scale bar, 25 *µ*m. **(H)** Quantification of (G): Oil Red O. Positive area, percentage area stained with Oil Red O and quantified as a percentage of the total area on eight random frames (magnification of ×40) per heart (n_heart_ = 3 [WT], 3 [MHC-*Plin5*]; n_frame_ = 24 [WT], 24 [MHC-*Plin5*]). **(I)** Triglyceride content in hearts from WT and MHC-*Plin5* mice, 4 h after fasting (n_heart_ = 6 [WT], 6 [MHC-*Plin5*]). **(J, K)** Lipid content in hearts from WT and MHC-Plin5 mice, 4 h after fasting. LPC, lysophosphatidylcholine; PS, phosphatidylserine; PC, phosphatidylcholine; PE, phosphatidylethanolamine; CE, cholesterol esters; DG, diacylglycerol; SM, sphingomyelin; Cer, ceramide; DiCer, dihydroceramide; GlcCer, glucosylceramide; LacCer, lactosylceramide (n_heart_ = 7 [WT], 7 [MHC-*Plin5*]). Values are the mean ± SEM; *P*-values are calculated by a *t* test. Source data are available for this figure.

To determine whether elevated Plin5 regulates cardiac contractility, we generated mice with cardiac-specific overexpression of FLAG-tagged *Plin5* (MHC-*Plin5* mice) (Fig S1A–C). The cardiac Plin5 protein levels in MHC-*Plin5* mice were increased within physiological range and comparable to up-regulation by fasting (Fig S1B–E). In addition, cardiac Plin5 showed a similar expression pattern in MHC-*Plin5* cardiomyocytes as in WT (Fig S1F). Lipid droplets and triglyceride levels were elevated in MHC-*Plin5* versus WT hearts, but other lipids in the hearts were unchanged between genotypes (Fig S1G–K). Body weight and the levels of circulating lipids, glucose, and insulin were not statistically different between the genotypes (Table S1). Echocardiography of 11-wk-old mice under baseline conditions showed that cardiac function and heart rate were comparable in WT and MHC-*Plin5* mice (Table 1). However, left ventricular mass was significantly higher in MHC-*Plin5* than in WT hearts, with a concomitant increase in relative wall thickness and posterior wall dimension (Table 1). Left ventricular end-diastolic dimension and volume were similar between groups, showing no apparent left ventricular enlargement of MHC-*Plin5* hearts. In addition, heart weight was significantly increased in MHC-*Plin5* (Table S2). Our findings are in accordance with previous reports showing that cardiomyocyte-specific overexpression of Plin5 promotes cardiac hypertrophy without affecting heart function (Wang et al, 2013; Kolleritsch et al, 2019).

Table S1 Metabolic and plasma parameters in WT and MHC-Plin5 mice.

**Table 1.** Cardiac Plin5 overexpression results in hypertrophic hearts but maintained heart function in 11-wk-old mice.

Parameters	WT (n = 9–10)	MHC-*Plin5* (n = 9)	*P*-value
Body weight (g)	26.4 ± 0.5	26.3 ± 0.6	0.898
LV mass (mg)	93.8 ± 4.7	111.4 ± 4.4	**0.015**
RWT	0.32 ± 0.01	0.36 ± 0.01	**0.034**
LVAWD(d) (mm)	0.74 ± 0.03	0.79 ± 0.03	0.275
LVPWD(d) (mm)	0.57 ± 0.02	0.68 ± 0.01	**<0.001**
LVED(d) (mm)	4.07 ± 0.08	4.13 ± 0.06	0.596
LVVol(d) (*µ*l)	72.0 ± 3.3	72.0 ± 3.0	0.997
Ejection fraction (%)	59.1 ± 1.3	57.5 ± 2.0	0.485
Stroke volume (*µ*l)	42.2 ± 1.2	41.4 ± 2.2	0.739
Heart rate (bpm)	405 ± 15	415 ± 8	0.585
Cardiac output (ml/min)	17.1 ± 0.9	17.2 ± 1.0	0.942

Cardiac morphological and functional parameters assessed by echocardiography in 11-wk-old WT and MHC-Plin5 mice. RWT, relative wall thickness; LVAWD(d), LV anterior wall dimension in diastole; LVPWD(d), LV posterior wall dimensions in diastole; LVED(d), LV end-diastolic dimension; and LVVol(d), LV end-diastolic volume LVED(d). Results are expressed as the mean ± SEM; *P*-values are calculated by a *t* test; significant *P*-values (<0.05) are shown in bold.

Table S2. Heart, liver, and lung weights in both young and naturally aging WT and MHC-Plin5 mice.

Given that contractile dysfunction is a hallmark of long-term hypertrophy and cardiac aging (Diwan & Dorn, 2007; Dong et al, 2020), we also investigated the effect of cardiac Plin5 overexpression in older (27-wk-old) mice. Heart weight was significantly increased in MHC-*Plin5* (Table S2). Echocardiography showed that left ventricular volume, cardiac output, and heart rate were similar between the genotypes but ejection fraction and stroke volume were higher in MHC-*Plin5* mice (Table 2). In addition, Doppler analysis of the 27-wk-old mice excluded diastolic dysfunction in these mice (Table 3). Weights of lung and liver were not different between genotypes (Table S2). Thus, cardiac Plin5 overexpression and associated cardiac hypertrophy do not lead to myocardial failure.

**Table 2.** Cardiac Plin5 overexpression does not lead to early myocardial failure in 27-wk-old mice.

Parameters	WT (n = 10)	MHC-*Plin5* (n = 9)	*P*-value
Body weight (g)	41.1 ± 1.3	38.9 ± 1.5	0.288
LV mass (mg)	130.0 ± 5.2	147.0 ± 10.8	0.162
RWT	0.43 ± 0.02	0.49 ± 0.03	0.110
LVAWD(d) (mm)	0.94 ± 0.03	1.04 ± 0.04	0.050
LVPWD(d) (mm)	0.77 ± 0.02	0.87 ± 0.07	0.194
LVED(d) (mm)	4.03 ± 0.11	3.95 ± 0.11	0.579
LVVol(d) (*µ*l)	72.1 ± 4.7	68.4 ± 4.7	0.590
Ejection fraction (%)	71.7 ± 2.6	80.6 ± 2.8	**0.033**
Stroke volume (*µ*l)	37.7 ± 1.9	46.1 ± 2.6	**0.018**
Heart rate (bpm)	414 ± 23	367 ± 28	0.209
Cardiac output (ml/min)	16.8 ± 1.7	16.9 ± 1.6	0.962

Cardiac morphological and functional parameters assessed by echocardiography in 27-wk-old WT and MHC-Plin5 mice. RWT, relative wall thickness; LVAWD(d), LV anterior wall dimension in diastole; LVPWD(d), LV posterior wall dimensions in diastole; LVED(d), LV end-diastolic dimension; and LVVol(d), LV end-diastolic volume LVED(d). Results are expressed as the mean ± SEM; *P*-values are calculated by a *t* test; significant *P*-values (<0.05) are shown in bold.

**Table 3.** Cardiac Plin5 overexpression does not lead to diastolic dysfunction in 27-wk-old mice.

Parameters	WT (n = 10)	MHC-*Plin5* (n = 7)	*P*-value
Body weight (g)	37.3 ± 1.2	38.8 ± 2.3	0.548
Heart rate (bpm)	465 ± 23	405 ± 20	0.077
DT (ms)	26.3 ± 1.6	31.1 ± 3.2	0.167
E (mm/s)	662.4 ± 18.4	605.9 ± 27.7	0.097
A (mm/s)	467.5 ± 33.1	487.1 ± 22.5	0.663
E/A ratio	1.46 ± 0.08	1.25 ± 0.05	0.060
E/E’ ratio	42.23 ± 2.99	46.50 ± 3.42	0.366
MPI	0.738 ± 0.027	0.721 ± 0.035	0.707

Echocardiographic parameters of left ventricular diastolic function in 27-wk-old WT and MHC-Plin5 mice. DT, deceleration time; and MPI, myocardial performance index. Results are expressed as the mean ± SEM; *P*-values are calculated by a *t* test.

### Cardiac *Plin5* overexpression does not affect fibrosis nor genes/protein markers involved in pathological hypertrophy

**Figure 2.**
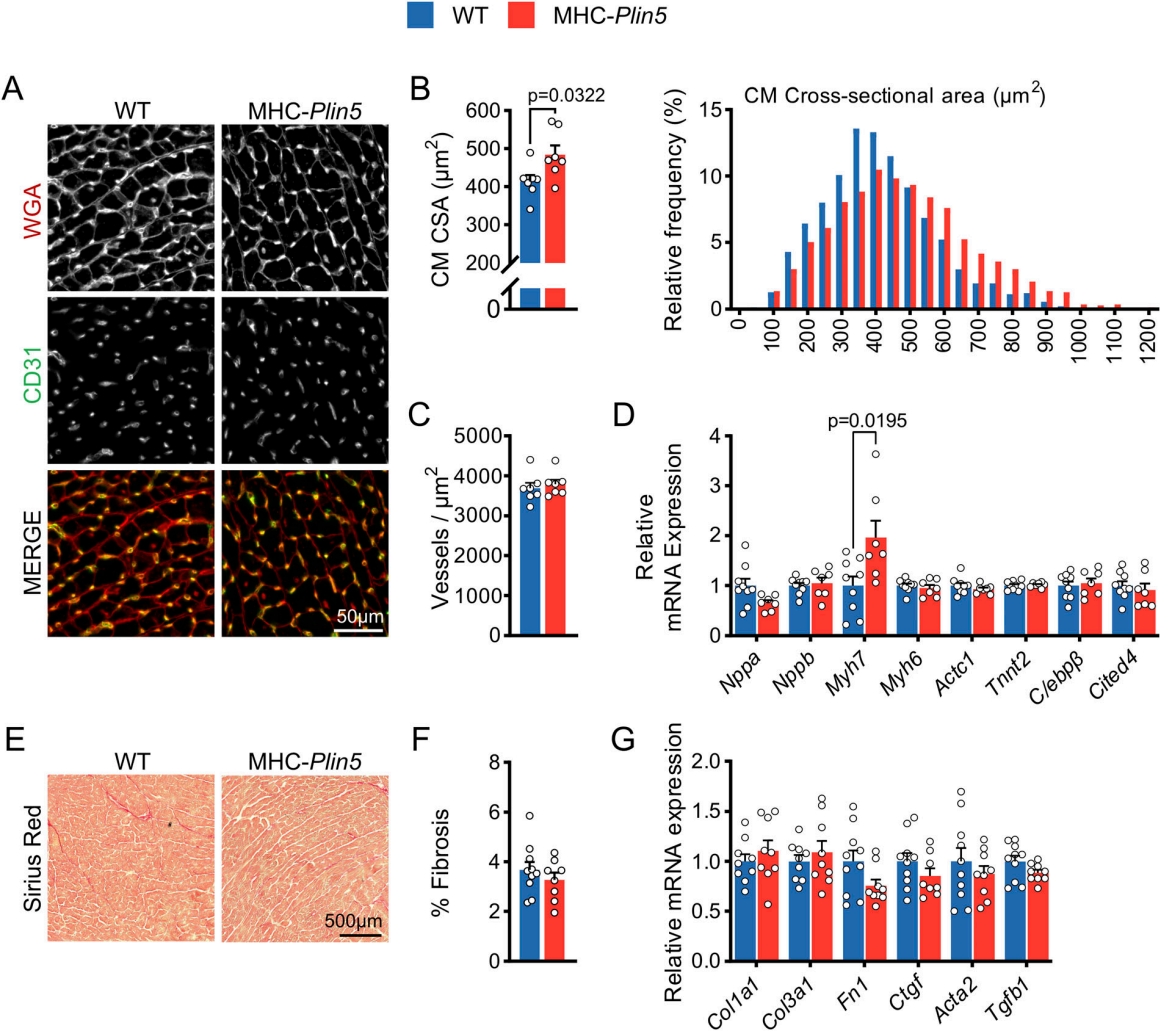
Markers of pathological hypertrophy are not up-regulated in MHC-*Plin5* cardiomyocytes. **(A)** Representative heart cryosections of 22-wk-old WT and MHC-*Plin5* mice stained with fluorescent WGA (red) and CD31 (green). Scale bar, 50 *µ*m. **(B)** Left, quantification of (A): cardiomyocyte cross-sectional area (CM CSA) (n_mice_ = 7 [WT], 7 [MHC-*Plin5*]). Right, distribution of CM CSA in all measured cardiomyocytes (calculated from n_mice_ = 7 [WT], 7 [MHC-Plin5]; n_cell_ = 1,400 [WT], 1,394 [MHC-*Plin5*]). **(C)** Quantification of (A): vessel density within cardiac tissues (n_mice_ = 7 [WT], 7 [MHC-*Plin5*]). **(D)** mRNA expression of the indicated genes in isolated primary cardiomyocytes from 27-wk-old WT and MHC-*Plin5* mice (n_mice_ = 9 [WT], 7 [MHC-*Plin5*] from ≥2 independent isolations). **(E)** Representative heart cryosections of 27-wk-old WT and MHC-*Plin5* mice stained with picrosirius red. Scale bar, 500 *µ*m. **(F)** Quantification of (E): % fibrosis, percentage area stained with picrosirius red quantified as a percentage of the total area of the heart cryosection (n_mice_ = 10 [WT], 8 [MHC-*Plin5*]). **(G)** mRNA expression of the indicated genes in heart from 27-wk-old WT and MHC-*Plin5* mice (n_mice_ = 9–10 [WT], 8–9 [MHC-*Plin5*]). Values are the mean ± SEM; *P*-values are calculated by a *t* test.

To clarify the nature of the cardiac hypertrophy in naturally aging mice, we stained cardiac sections with WGA and found that cardiomyocyte cross-sectional area was significantly higher in MHC-*Plin5* hearts than in WT hearts (Fig 2A and B). Given that preservation of vascular density is an important aspect of beneficial hypertrophy, we assessed cardiac vascular density by CD31 staining and showed that it was similar in MHC-*Plin5* and WT hearts (Fig 2A and C). Considering the significant increase observed in cardiomyocyte cross-sectional area, these data suggest maintained or increased capillary-to-myocyte ratio in MHC-*Plin5* hearts. Thus, naturally aging Plin5-overexpressing hearts exhibit capillary density matched to the degree of myocardial hypertrophy (i.e., capillary density is maintained in the normal range).

Plin5 has previously been shown to function as a transcriptional co-activator, mainly promoting transcription of genes that mediate mitochondrial biogenesis and oxidative function (Gallardo-Montejano et al, 2016). Here, we analyzed gene expression in primary cardiomyocytes isolated from MHC-*Plin5* and WT mice and observed a significant up-regulation of the *Myh7* gene involved in cardiac hypertrophy, in MHC-*Plin5* cardiomyocytes (Fig 2D). However, fetal genes such as *Nppa*, *Nppb*, and the fetal isoform of the contractile protein skeletal α-actin (*Actc1*), which are generally induced by pathological cardiac hypertrophy (Nakamura & Sadoshima, 2018), were not up-regulated (Fig 2D). In addition, fibrosis in mouse hearts, assessed using picrosirius red and molecular markers of fibrosis, was not altered by cardiac *Plin5* overexpression (Fig 2E–G).

### Physiological hypertrophy of MHC-*Plin5* hearts occurs independently of the canonical Akt/mTOR pathway

**Figure S2.**
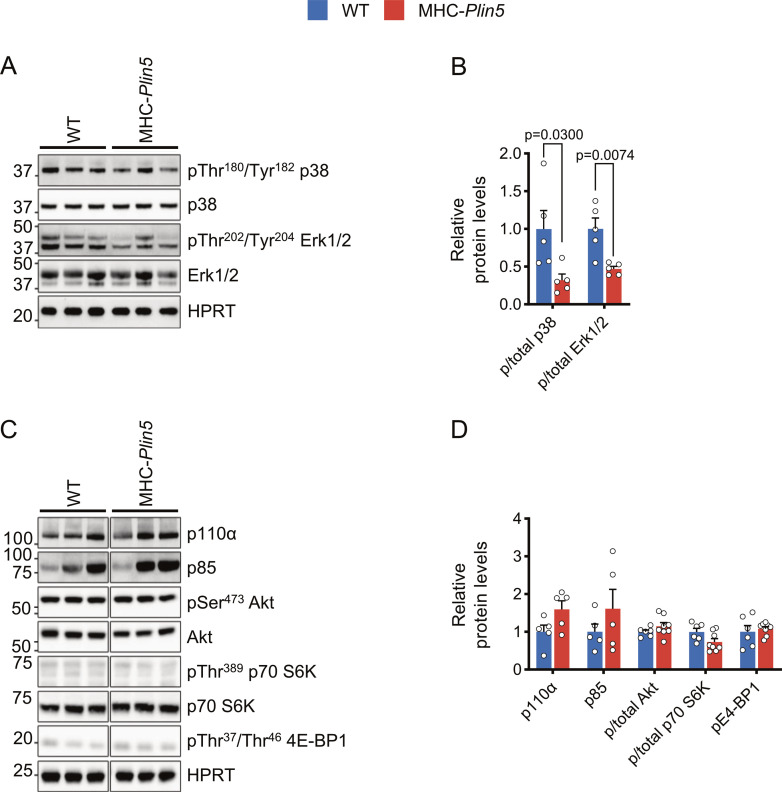
Intracellular signaling pathways regulating cardiac growth in MHC-*Plin5* cardiomyocytes. **(A)** Representative immunoblot images of p38 (pThr^180^/Tyr^182^ and total) and Erk1/2 (pThr^202^/Tyr^204^ and total) in MHC-*Plin5* and WT heart lysates after a 16-h fast. HPRT, loading control. **(B)** Quantification of (A). **(C)** Representative immunoblot images of p110α, p85, Akt (pSer^473^ and total), p70 S6K (pThr^389^ and total), and E4-BP1 (pThr^37^/Thr^46^) in MHC-*Plin5* and WT heart lysates after a 16-h fast. **(D)** Quantification of (C). Values are the mean ± SEM; *P*-values are calculated by a *t* test (n = 5–6 [WT], 5–8 [MHC-*Plin5*]). Source data are available for this figure.

We next assessed whether *Plin5* overexpression affected intracellular signaling pathways regulating cardiac growth. MAPK activation is associated with the development of pressure overload–induced pathological hypertrophy (Zhang et al, 2003), but we found significant down-regulation of p-ERK1/2 and p-p38 in MHC-*Plin5* mouse hearts (Fig S2A and B). Signal transduction pathways critical for myosin growth also include the PI3K/Akt/mammalian target of rapamycin (mTOR) pathway, which has been suggested to discriminate physiological from pathological hypertrophy (Kemi et al, 2008). We found small but non-significant increases in the PI3K subunits p110α and p85 in MHC-*Plin5* mouse hearts (Fig S2C and D). Total Akt protein level and its serine 473 phosphorylation were not different between genotypes (Fig S2C and D). In addition, activation of mTOR, measured as phosphorylation of its two substrates, the ribosomal protein S6 kinase 1 and the eukaryotic translation initiation factor 4E–binding protein 1 (4E-BP1), was unchanged in MHC-*Plin5* hearts. These results indicate that physiological hypertrophy occurs independently of the canonical Akt/mTOR pathway.

### The cardiac Plin5 interactome reveals an interaction between Plin5 and SERCA2

**Figure 3.**
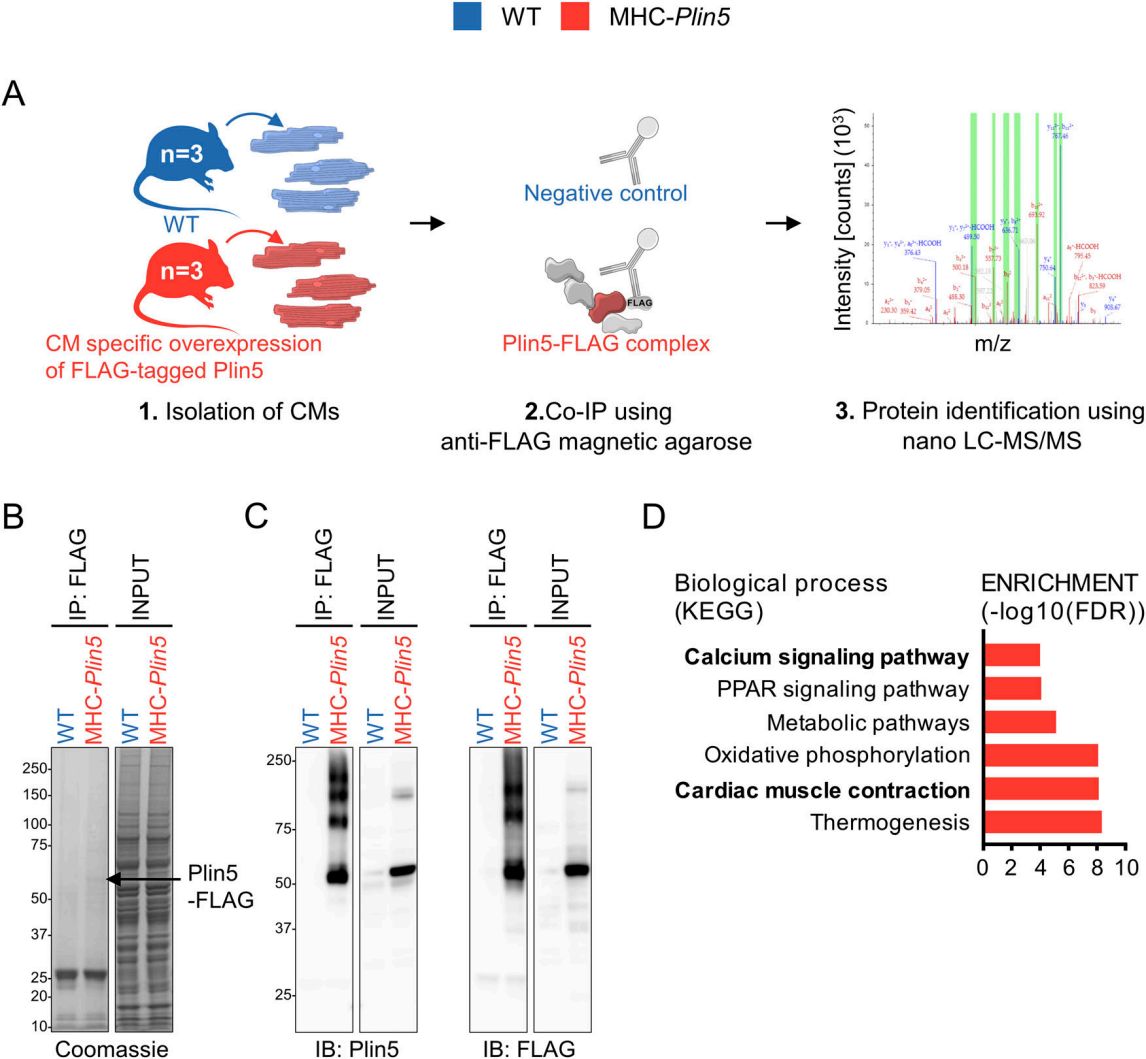
Interactome reveals SERCA2 as a novel partner of Plin5. **(A)** Experimental approach. CM, cardiomyocyte. Nano-LC–MS/MS, nanoscale liquid chromatography coupled to tandem mass spectrometry. **(B)** Coomassie blue–stained gels showing FLAG-enriched proteins (IP: FLAG) and total cell lysate (INPUT) from WT and MHC-*Plin5* cardiomyocytes. Black arrow indicates the assumed Plin5-FLAG protein. **(C)** Representative immunoblot images of Plin5 (left) and FLAG (right) in FLAG-enriched and total fractions. **(D)** List of significantly enriched KEGG pathways in FLAG-enriched fraction of MHC-*Plin5* versus WT cardiomyocytes. Source data are available for this figure.

**Figure 4.**
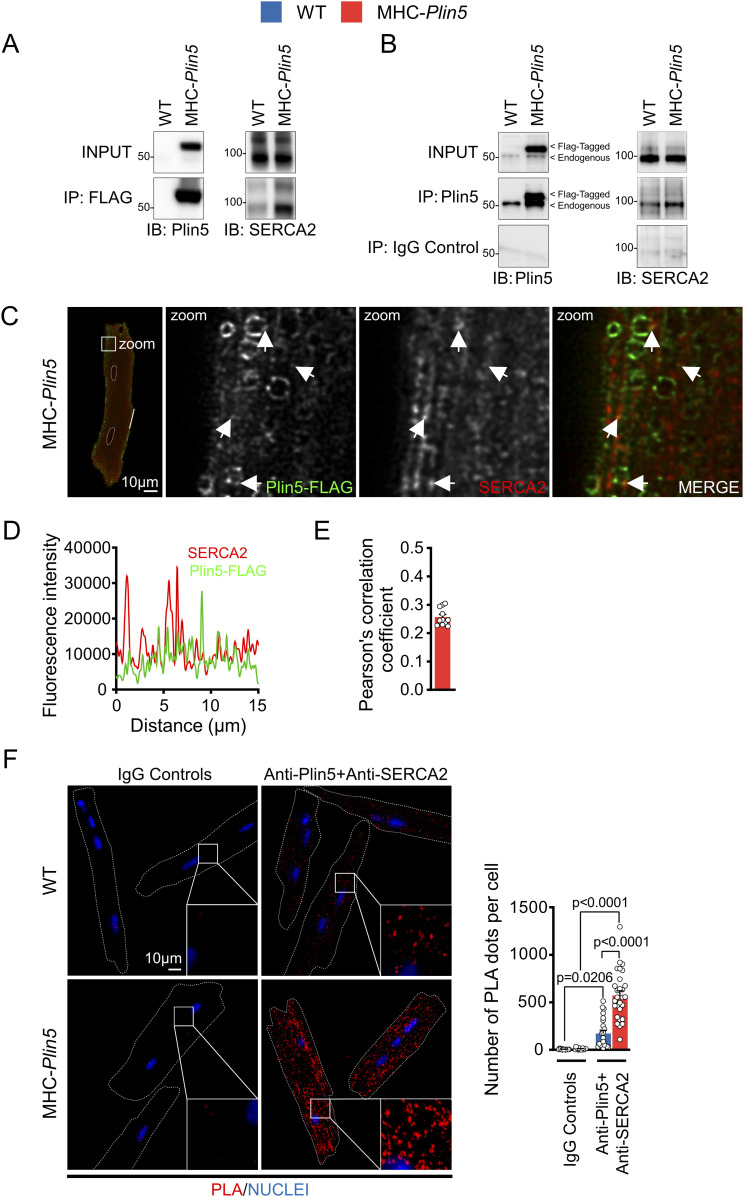
Plin5 and SERCA2 interact in both WT and MHC-*Plin5* cardiomyocytes. **(A)** Representative immunoblot images of Plin5 (left) and SERCA2 (right) in total cell lysates (INPUT) and FLAG co-immunoprecipitates from WT and MHC-*Plin5* cardiomyocytes (results shown are representative of n_mice_ = 4 [WT], 3 [MHC-Plin5]). **(B)** Representative immunoblot images of Plin5 (left) and SERCA2 (right) in total cell lysates (INPUT) and Plin5 co-immunoprecipitates from WT and MHC-Plin5 cardiomyocytes. Non-immune rabbit IgG (IgG control) was used as the IP-negative control (results shown are representative of n_mice_ = 4 [WT], 6 [MHC-*Plin5*] from two independent isolations). **(C)** Representative single z-plane confocal scan of immunofluorescent staining of an MHC-*Plin5* cardiomyocyte. FLAG-Plin5 signal is green, SERCA2 signal is red, and the white arrows indicate colocalization. Scale bar, 10 *µ*m. **(C, D)** Fluorescence intensity plot of the distribution of fluorescence from the MHC-*Plin5* cardiomyocyte across white line in (C). **(E)** Quantitative analysis of FLAG-Plin5 and SERCA2 colocalization analyzed by Pearson’s correlation coefficient (calculated from n_mice_ = 2 [MHC-*Plin5*]; n_cell_ = 11). **(F)** Left, representative images of in situ interactions between Plin5 and SERCA2 using in situ proximity ligation assay (PLA) in adult WT and MHC-*Plin5* cardiomyocytes. Nuclei were stained using DRAQ5. PLA signal is red, and DRAQ5 signal is blue. Non-immune rabbit IgG and mouse IgG1 (IgG controls) were used as the biological negative control. Right, quantification of the PLA fluorescent dots (n_mice_ = 2 [WT], 2 [MHC-*Plin5*]; n_cell_ = 16 [WT-IgG controls], 15 [MHC-*Plin5*-IgG controls], 23 [WT-anti-Plin5 + anti-SERCA2], 28 [MHC-*Plin5*-anti-Plin5 + anti-SERCA2]). Values are the mean ± SEM; *P*-values are calculated by two-way ANOVA followed by Tukey’s multiple comparisons post hoc test. Source data are available for this figure.

**Figure S3.**
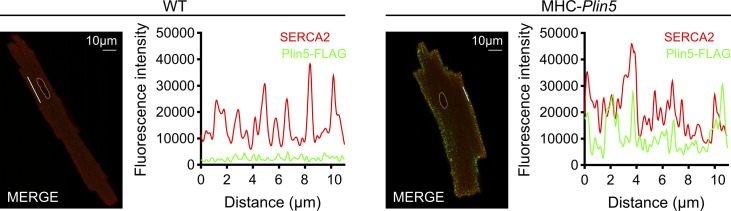
Colocalization of Plin5 and SERCA2 in MHC-*Plin5* cardiomyocytes. Representative confocal images of the subcellular localization of FLAG-Plin5 (green) and SERCA2 (red) and respective fluorescence intensity plots in WT and MHC-*Plin5* cardiomyocytes. Scale bar, 10 *µ*m.

**Figure S4.**
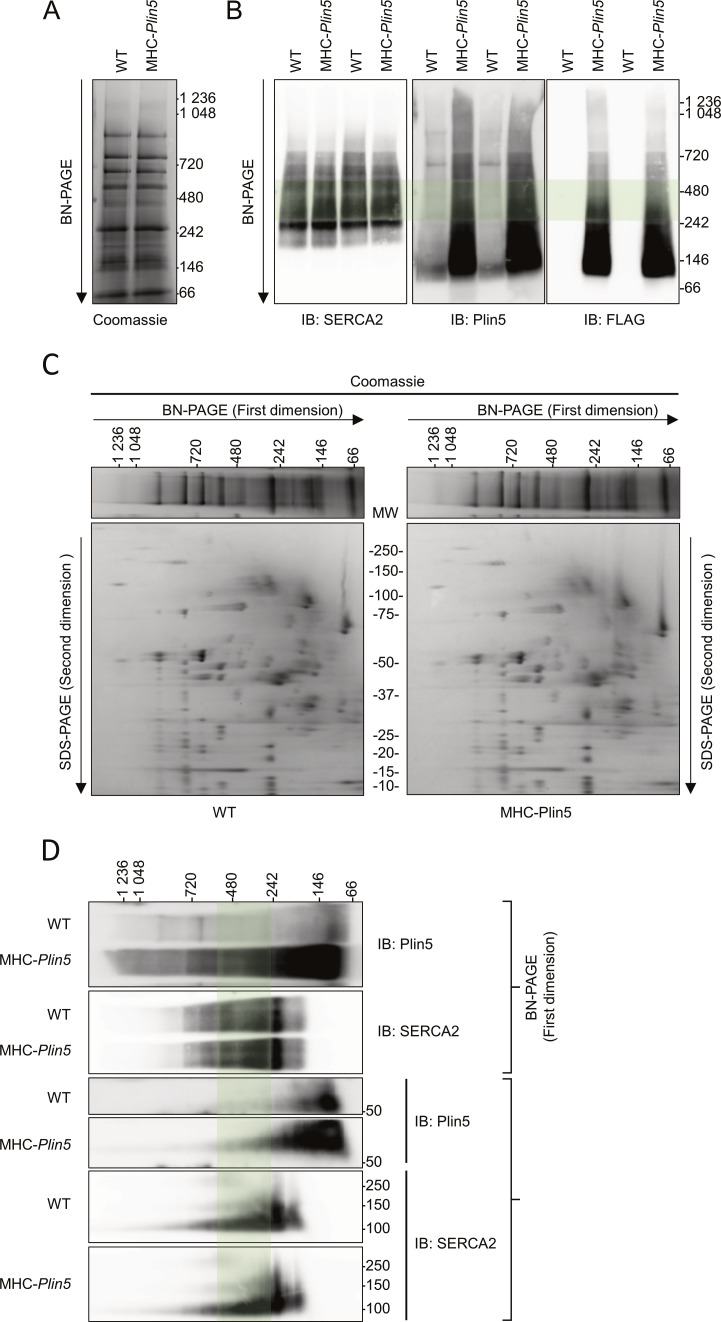
Two-dimensional blue-native/SDS–PAGE for complex analysis of Plin5 and SERCA2 in WT and MHC-*Plin5* mice. **(A)** Coomassie staining of blue-native PAGE of WT and MHC-*Plin5* primary cardiomyocytes. **(A, B)** Proteins were separated as in (A) and analyzed by immunoblotting with the indicated antibodies. Green shading highlights the macrocomplex that contained both Plin5 and SERCA2. **(C)** Coomassie staining of two-dimensional blue-native/SDS–PAGE of WT and MHC-*Plin5* primary cardiomyocytes. The first horizontal dimension separated protein complexes according to their molecular sizes, and the second vertical dimension displayed each component of the complexes. MW, molecular weight. **(C, D)** Proteins were separated as in (C) and analyzed by immunoblotting with the indicated antibodies. Green shading highlights the macrocomplex that contained both Plin5 and SERCA2 (results shown are representative of n_mice_ = 3 [WT], 3 [MHC-Plin5] from two independent isolations). Source data are available for this figure.

To elucidate how Plin5 induces physiological cardiac hypertrophy, we used quantitative proteomics to identify potential binding partners of Plin5. We performed co-immunoprecipitation experiments coupled with mass spectrometry on protein lysates from primary MHC-*Plin5* and WT cardiomyocytes, using the FLAG antibody to detect Plin5 (Fig 3A–C). A total of 407 proteins were identified in the FLAG-enriched fraction; of these, 79 were present at a >twofold higher level in lysates from MHC-*Plin5* versus WT cardiomyocytes (data are available via ProteomeXchange with identifier PXD035121). KEGG pathway enrichment analyses (Fig 3D) were conducted using ShinyGO (Ge et al, 2020) and revealed that the majority of the thirty most significantly enriched pathways in the FLAG-enriched fraction of MHC-*Plin5* versus WT cardiomyocytes included known functions of Plin5, namely, oxidative phosphorylation, fatty acid degradation, and PPAR signaling (Mason & Watt, 2015; Najt et al, 2020; Gallardo-Montejano et al, 2021). Cardiac muscle contraction and calcium handling were also recognized as enriched pathways of the Plin5 interactome. Sarcoplasmic/endoplasmic reticulum Ca^2+^ ATPase 2 (SERCA2) was identified as one of the strongest Plin5-interacting candidates participating in the enrichment of these pathways, with a high number of identified peptides and good coverage (30%). Independent co-immunoprecipitation experiments of FLAG-Plin5 on protein lysates from primary MHC-*Plin5* and WT cardiomyocytes confirmed that overexpressed Plin5 and SERCA2 form a complex in MHC-*Plin5* cardiomyocytes (Fig 4A). We could confirm the interaction between PLIN5 and SERCA2 in MHC-*Plin5* cardiomyocytes using an anti-Plin5 antibody (Fig 4B). Importantly, we could also detect this interaction between endogenous PLIN5 and SERCA in WT cardiomyocytes (Fig 4B). In addition, using confocal microscopy we showed a partial colocalization of Plin5 and SERCA2 in MHC-*Plin5* cardiomyocytes (Figs 4C–E and S3). To further confirm the Plin5/SERCA interaction, we also performed an in situ proximity ligation assay. Importantly, we detected a specific interaction between Plin5 and SERCA2 in both WT- and Plin5-overexpressing cardiomyocytes (Fig 4F). The formation of Plin5/SERCA2 protein complexes was significantly higher in the Plin5-overexpressing cardiomyocytes (Fig 4F). In addition, using two-dimensional blue-native PAGE separation on cardiomyocyte homogenates, we found that endogenous Plin5 and overexpressed Plin5 were present in similar size complexes (Fig S4). Notably, Plin5 was present in complexes encompassing SERCA2 in both WT and MHC-*Plin5* mice (Fig S4).

An earlier study in human AC16 cardiomyocytes reported Plin5 interactome data (Kien et al, 2022). The authors focused on mitochondrial partners of Plin5, and they did not report any interactions with Ca^2+^ signaling proteins. However, by exploring their raw dataset available online, we could identify SERCA2 as a binding partner of Plin5, strengthening the evidence for a Plin5/SERCA2 interaction in cardiomyocytes.

Further work is needed to determine whether the interaction between Plin5 and SERCA2 occurs at lipid droplet–SR contact sites or whether it results from a lipid droplet–independent SR anchoring of Plin5. Plin5 has been reported to localize to several intracellular locations and to the lipid droplet in a dot-like fashion, with abundance at lipid droplet–mitochondria tethering sites (Gemmink et al, 2018; Keenan et al, 2021). Unlike some lipid-droplet proteins, Plin5 does not have signal peptides for ER targeting, but it has been shown to be recruited to the ER when diacylglycerol levels are increased (Skinner et al, 2009).

### Enhanced Ca^2+^ handling through increased SERCA2 function in MHC-*Plin5* cardiomyocytes

SERCA2 is crucial for regulating the reuptake of Ca^2+^ into the SR to relax cardiomyocytes, making it a key protein for Ca^2+^ homeostasis and contractility in these cells (Zhihao et al, 2020). SERCA2 is composed of 10 transmembrane helices, which control Ca^2+^ binding and translocation, and a large cytoplasmic headpiece, which regulates ATP binding, autophosphorylation, and dephosphorylation. SERCA2 activity is regulated in a highly dynamic fashion through interaction with several transmembrane micropeptides, including phospholamban (PLN) (James et al, 1989; Kranias & Hajjar, 2012), sarcolipin (Autry et al, 2016), and dwarf (Fisher et al, 2021) open reading frame. Binding of non–ER-resident proteins to SERCA2 has been shown to modulate SERCA2 protein levels and/or activity through direct interaction with the protein. It is thus possible that the Plin5/SERCA2 interaction sterically contributes to modulation of SERCA2 function.

**Figure 5.**
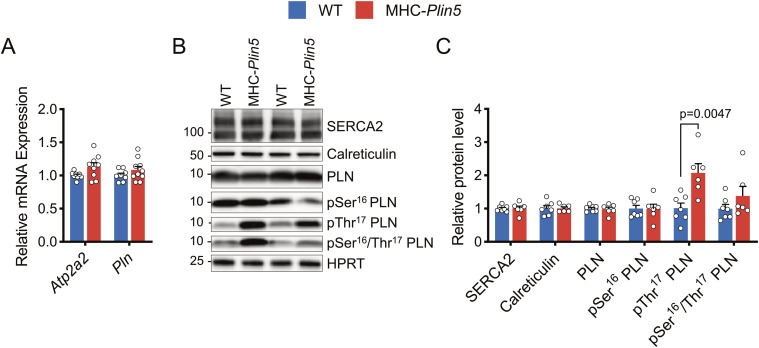
*Plin5* overexpression likely contributes to modulations of SERCA2 interaction capacity and/or modulation of regulatory partners. **(A)** mRNA expression of the indicated genes in isolated primary cardiomyocytes from WT and MHC-*Plin5* mice (n_mice_ = 8 [WT], 9–10 [MHC-*Plin5*] from ≥3 independent isolations). **(B)** Representative immunoblot images of SERCA2, calreticulin, and PLN (pSer16, pThr17, pSer16/Thr17, and total) in isolated primary cardiomyocytes from WT and MHC-*Plin5* mice. HPRT served as a loading control. **(C)** Quantification of (B) (n_mice_ = 7 [WT], 6 [MHC-*Plin5*] from ≥2 independent isolations). Values are the mean ± SEM; *P*-values are calculated by a *t* test. Source data are available for this figure.

We first investigated whether Plin5 overexpression affected expression and/or regulation of SERCA2, with potential consequences for Ca^2+^ handling. mRNA and protein levels of SERCA2 were similar between MHC-*Plin5* and WT cardiomyocytes (Fig 5A–C). mRNA levels of the SERCA2 regulator PLN were also unchanged between the genotypes (Fig 5A). PLN is regulated by phosphorylation at Ser16 by cAMP-dependent PKA and at Thr17 by Ca^2+^/calmodulin-dependent protein kinase II (CaMKII) (Mattiazzi & Kranias, 2014). PLN phosphorylation dissociates the functional interactions of the PLN/SERCA2 complex, thereby releasing the allosteric inhibition of its activity (MacLennan & Kranias, 2003). Although total and Ser16 phosphorylated PLN protein levels were similar between WT and MHC-*Plin5* cardiomyocytes, Thr17 phosphorylation levels of PLN were significantly higher in MHC-*Plin5* cardiomyocytes (Fig 5B and C). These findings suggest that increased Plin5 levels modulate SERCA2 function.

**Figure 6.**
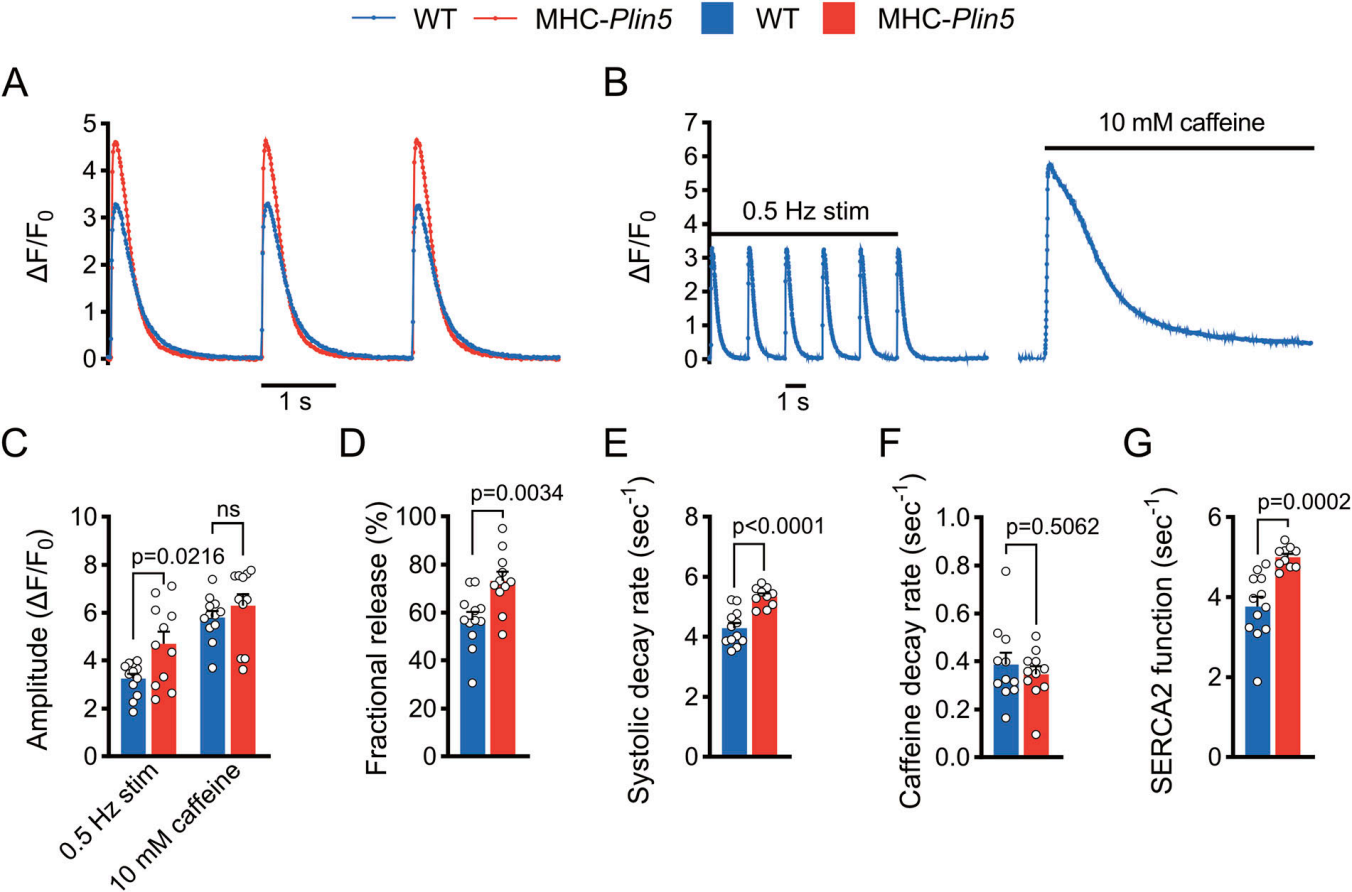
*Plin5* overexpression results in increased SERCA2 function. **(A)** Representative Ca^2+^ transients recorded in paced Fluo-4-AM–loaded primary cardiomyocytes from WT and MHC-*Plin5* mice. **(B)** Representative Ca^2+^ transients recorded in Fluo-4-AM–loaded primary cardiomyocytes from WT and MHC-*Plin5* mice after pacing or caffeine (10 mM) induction. Stim, stimulation. **(C, D, E, F, G)** Quantification (n_heart_ = 5 [WT], 5 [MHC-*Plin5*]; n_cell_ = 12 [WT], 11 [MHC-*Plin5*]) of (C) Ca^2+^ transient amplitude of paced and caffeine-induced WT and MHC-*Plin5* cardiomyocytes, (D) fractional release of Ca^2+^ during systolic contraction, (E) Ca^2+^ decay rates during systole in paced cardiomyocytes, (F) Ca^2+^ decay rates in the presence of caffeine, and (G) calculated contribution of SERCA2 activity for Ca^2+^ removal during relaxation (SERCA2 function). **(C, D, E, F, G)** Values are the mean ± SEM; *P*-values are calculated by one-way ANOVA followed by Sidak’s multiple comparisons post hoc tests for (C) and a *t* test for (D, E, F, –G).

**Figure S5.**
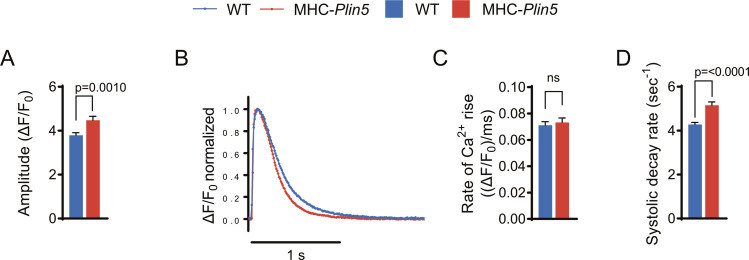
Ca^2+^ handling in paced single-intact cardiomyocytes isolated from WT and MHC-*Plin5* mice. **(A, B, C)** Quantification of Ca^2+^ transients recorded in paced Fluo-4-AM–loaded primary cardiomyocytes from WT and MHC-*Plin5* mice. **(A)** Ca^2+^ transient amplitude. **(B)** Representative normalized confocal Ca^2+^ transient from WT and MHC-*Plin5* cardiomyocytes. **(C, D)** Rate of Ca^2+^ rise and (D) rate of Ca^2+^ removal. Values are the mean ± SEM; *P*-values are calculated by a *t* test (n_heart_ = 5 [WT], 4 [MHC-*Plin5*]; n_cell_ = 65 [WT], 45 [MHC-*Plin5*]).

**Figure S6.**
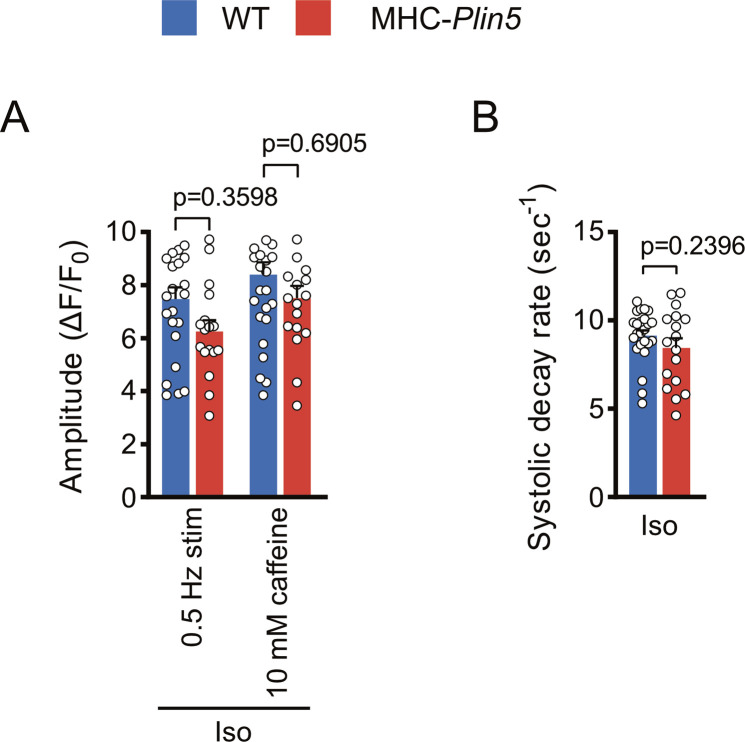
Ca^2+^ handling in isoproterenol (Iso)-stimulated cardiomyocytes isolated from WT and MHC-*Plin5* mice. **(A, B)** Quantification of (A) Ca^2+^ transient amplitude of paced and caffeine-induced 10 nM Iso-stimulated WT and MHC-*Plin5* cardiomyocytes (n_heart_ = 4 [WT], 3 [MHC-*Plin5*]; n_cell_ = 24–27 [WT], 17–18 [MHC-*Plin5*]) and (B) Ca^2+^ decay rates during systole in paced 10 nM Iso-stimulated cardiomyocytes (n_heart_ = 4 [WT], 3 [MHC-*Plin5*]; n_cell_ = 24 [WT], 17 [MHC-*Plin5*]). **(A, B)** Values are the mean ± SEM; *P*-values are calculated by one-way ANOVA followed by Sidak’s multiple comparisons post hoc tests for (A) and a *t* test for (B).

To determine whether Plin5 overexpression alters Ca^2+^ homeostasis in cardiomyocytes, we used live-cell imaging to assess intracellular Ca^2+^ cycling in paced MHC-*Plin5* and WT cardiomyocytes loaded with the Ca^2+^ indicator Fluo-4. After electrical stimulation, the peak amplitude of Ca^2+^ transients was significantly higher in MHC-*Plin5* than in WT cardiomyocytes (Figs 6A and S5A). The rate of Ca^2+^ rise was unchanged between genotypes, but MHC-*Plin5* cardiomyocytes displayed faster decay kinetics compared with WT cardiomyocytes (Fig S5B–D). As the amplitude of a Ca^2+^ transient depends on Ca^2+^ reuptake by the SR, we assessed the SR Ca^2+^ content in MHC-*Plin5* and WT cardiomyocytes by triggering maximal Ca^2+^ release with rapid caffeine application after electrical stimulation (Fig 6B). The SR Ca^2+^ content was similar between genotypes (Fig 6C). However, the fractional Ca^2+^ release from the SR, as determined by the ratio of Ca^2+^ released during electrical stimulation to Ca^2+^ released during caffeine application, was significantly higher in MHC-*Plin5* cardiomyocytes (Fig 6D). Next, we stimulated cardiomyocyte contraction with the β-adrenergic receptor agonist isoproterenol. As expected, the peak amplitude of Ca^2+^ transients and systolic decay rate were markedly elevated in both WT and MHC-*Plin5* cardiomyocytes compared with baseline (Fig S6A and B). However, there were no differences between genotypes (Fig S6A and B).

The faster decay kinetics of MHC-*Plin5* cardiomyocytes could be explained either by increased extrusion of Ca^2+^ across the sarcolemma, which occurs predominantly via the Na^+^/Ca^2+^ exchanger (NCX) (Díaz et al, 2004), or by enhanced SERCA2 pumping of Ca^2+^ into the SR. To discriminate between these two possibilities, we compared the decay rate of the systolic Ca^2+^ transient, which depends on NCX and SERCA2 activity, with the decay rate of the caffeine-induced Ca^2+^ transient, which depends almost exclusively on NCX. Although the systolic decay rate was higher in MHC-*Plin5* cardiomyocytes than in WT cells (Fig 6E), the caffeine-induced decay rate was not different between genotypes (Fig 6F), arguing against an alteration in NCX activity. However, the contribution of SERCA2 activity to Ca^2+^ removal, that is, SERCA2 function (calculated by subtracting the two decay rates [Hu et al, 2017]), was significantly higher in MHC-*Plin5* cardiomyocytes (Fig 6G).

Collectively, our results show that elevated cardiac Plin5 levels are beneficial and promote physiological hypertrophy. Furthermore, our findings highlight Plin5 as an important player in the regulation of cardiac contractility and Ca^2+^ handling through modification of SERCA2 function. Modulation of cardiac Plin5 levels may thus provide a novel therapeutic strategy to improve cardiac contractility.

## Materials and Methods

### Analysis of human RNA-seq data

Human RNA-seq data were obtained from the Genotype-Tissue Expression project portal on 02/08/2022. We used the *PLIN5* TPM values from the left ventricle to stratify the data into low and high *PLIN5* groups, based on the bottom and top quartiles, respectively. We regarded data outside the 1.5 times interquartile range as outliers and removed them for the subsequent analyses. Subsequently, we performed differential expression and functional analyses between the selected groups using the method described previously (Arif et al, 2021).

### Mice

All animal procedures performed were approved by the local animal ethics committee on animal experiments in Gothenburg (breeding ethics approval reference no. 1124-2017 and myocardial infarction ethics approval reference no. 2121-2019) and conform to the guidelines from Directive 2010/63/EU of the European Parliament on the protection of animals used for scientific purposes. To avoid the impact of sexual dimorphism in the study traits, only male mice have been used in this study. Unless otherwise stated, all mice used are 11-wk-old (considered young adult mice). At the end of experiments, mice were killed by cervical dislocation in an overdose of isoflurane anesthesia. Transgenic mice (MHC-*Plin5*) expressing FLAG-tagged mouse *Plin5* cDNA under the control of the cardiomyocyte-specific α-myosin heavy chain (MHC, *Myh6*) promoter were created by cloning mouse *Plin5* cDNA (*Lsdp5*, GenBank accession number BC024138.1) and a 3xFLAG epitope into a mouse *Myh6* vector. The transgene was introduced into C57BL/6NTac fertilized eggs by microinjection (done at Taconic Artemis). Founder animals were bred with C57BL/6N mice for five generations, and an inbred transgenic mouse line was established. WT and MHC-*Plin5* littermates were housed in a pathogen-free, temperature-controlled barrier facility (12-h light/12-h dark cycle) and fed rodent chow diet.

### Lipid analyses of heart tissue

Heart tissue was excised from WT and MHC-*Plin5* mice after a 4-h fast. The atrium of the heart was cut out, and the ventricles were quickly washed in PBS and thereafter snap-frozen in liquid nitrogen. 50–100 mg tissue was homogenized in methanol using a combination of a Precellys 24 homogenizer (Bertin Technologies) and Mixer/Mill equipment (Retsch). Lipids from the homogenized tissue were extracted using the Folch procedure (Folch et al, 1957). Heptadecanoyl (C17:0)-containing internal standards were added during the extraction. The extracts were evaporated using nitrogen, reconstituted in chloroform:methanol [2:1], and stored at −20°C until further analysis. Cholesteryl esters, triglycerides, diglycerides, phospholipids, and sphingomyelin were quantified using direct infusion/mass spectrometry according to a previous publication (Ståhlman et al, 2013). Ceramides, dihydroceramides, glucosylceramides, and lactosylceramides were quantified using ultraperformance liquid chromatography–tandem mass spectrometry (UPLCMS/MS) (Amrutkar et al, 2015).

### Quantitative immunohistochemistry analyses

Mouse hearts were embedded in OCT Cryomount (Histolab Products), frozen in liquid nitrogen–cooled isopentane, and cut into 10-μm-thick cross sections. Heart cryosections were stained with picrosirius red for the detection of fibrosis or Oil Red O (C_26_H_24_N_4_O) to detect and analyze neutral lipids. For fibrosis assessment, heart sections were fixed in 10% formaldehyde without methanol for 5 min, rinsed in running tap water, stained in 0.1% picrosirius red solution for 1 h, and washed twice in acidified water. The sections were dehydrated in ethanol (70% and 95% for 30 s each, and 100% for 2 min), cleared in TissueClear solution, and mounted in a resinous medium (Eukitt; Sigma-Aldrich). Fibrosis was measured with ImageJ as the picrosirius red–stained area on images covering the entire heart and expressed as percent of total area. For Oil Red O analysis, sections were stained according to a method described previously (Mehlem et al, 2013). Oil Red O–positive area was measured with ImageJ on eight frames (magnification of ×40) per heart and expressed as percent of total area of each frame. High-resolution images were obtained with a Mirax digital slide scanner (Carl Zeiss) for analysis.

### Echocardiography in mice

Echocardiography was performed at baseline and after an intraperitoneal injection of low-dose dobutamine (2 *µ*g/g body weight) in 11- or 27-wk-old isoflurane-anesthetized (1.2%) male mice; chest hair was removed with gel, the mouse was placed on a heating pad, and extremities were connected to an ECG electrode. Echocardiography was done with a VisualSonics Vevo 2100 system and an ultra-high-frequency linear array transducer (MS550D; VisualSonics). An optimal parasternal long-axis cine loop was acquired at >1,000 frames/s with the ECG-gated kilohertz visualization technique. Parasternal short-axis cine loops were acquired at 1, 3, and 5 mm below the mitral annulus. End-diastolic and end-systolic LV volumes and EF were calculated by biplane Simpson’s formula using the three parasternal short-axis views and the parasternal long-axis view. M-mode measurements at the 3-mm level were done with the leading-edge method. End-diastole was defined at the onset of the QRS complex, and end-systole, as the time of peak inward motion of the interventricular septum. At least three beats were averaged for each measurement. LV diastolic function was measured by analyzing the characteristic flow profile of the mitral valve Doppler and tissue Doppler flow, which was visualized in an apical four-chamber view. The myocardial performance index (Tei et al, 1997), which is an objective parameter incorporating systolic and diastolic time intervals and defines global systolic and diastolic ventricular function, was calculated as the sum of IVRT and IVCT divided by aortic ejection time (Arnlov et al, 2004). WT and MHC-*Plin5* mice of the same age were matched and compared within the same experiment. The echocardiographic examination was completed within 30 min after anesthetizing the mouse, and each echocardiographic examination was performed by an experienced echocardiographer. Evaluation of the stored data was performed offline in a blinded fashion using the Vevo Lab software system (VisualSonics).

### Immunofluorescence

Heart cryosections (10 *µ*m) were stained by fluorescent WGA and anti-CD31 (AF3628 from R&D Systems) according to standard methods. Myocyte cross-sectional area was quantified using ImageJ software as described (Tronchere et al, 2017). Primary cardiomyocytes were stained by anti-Plin5 (26951-1-AP from ProteinTech) according to standard methods. For Plin5 and SERCA2 colocalization, primary cardiomyocytes were seeded on glass coverslips in a plating medium for 2 h and fixed in 4% paraformaldehyde for 15 min. After permeabilization in 0.1% Triton X-100 in PBS for 5 min and blocking, the cells were probed with mouse anti-FLAG M2 antibody (F1804; Sigma-Aldrich) and rabbit anti-ATP2A2/SERCA2 antibody (9580; Cell Signaling Technology) for 16 h at 4°C. Cells were then probed with anti-rabbit Alexa Fluor 647–conjugated and anti-mouse Alexa Fluor 488–conjugated secondary antibodies and mounted in ProLongTM Glass Antifade Mountant (P36984; Invitrogen). Confocal images of WT and MHC-*Plin5* cardiomyocytes were acquired with 63.0× objective lens with oil immersion using Leica TCS-SP5 with Leica LAS AF software (Leica Microsystems) using a pinhole of 1 Airy unit. One confocal z-plane comprising the entire cardiomyocyte and encompassing the nucleus plane was acquired for each cell. Images were sampled according to Nyquist criteria and deconvoluted using Huygens Essential software, thereby optimizing images for accurate colocalization of fluorescent signals. For colocalization analysis, the threshold of each channel used to quantify colocalization was automatically determined using the method of Costes. Pearson’s coefficient, calculated with Huygens Essential software, was used to analyze colocalization.

### Isolation of primary cardiomyocytes

Hearts were rapidly excised from mice anesthetized with N-isoflurane. The aorta was cannulated, and the heart was perfused retrogradely with a Langendorff system (PanLab): first with perfusion buffer alone (120.4 mM NaCl, 14.7 mM KCl, 0.6 mM KH_2_PO_4_, 0.6 mM Na_2_HPO_4_, 1.2 mM MgSO_4_, 10 mM Na-Hepes, 5.5 mM glucose, 4.6 mM NaHCO_3_, 30 mM taurine, and 10 mM BDM, pH 7) for 4 min; then with perfusion buffer and collagenase type 2 (Worthington) for 3 min; and finally with digestion buffer supplemented with 40 mM CaCl_2_ for 8 min. After collagenase inhibition with fetal calf serum, the heart was torn apart, and cardiomyocytes were separated from non-cardiomyocytes by centrifugation at 20*g* for 3 min; the supernatant containing non-cardiomyocytes was used for fibroblast isolation. Cardiomyocytes were resuspended in perfusion buffer supplemented with 10% fetal calf serum and 12.5 *µ*M CaCl_2_. Thereafter, Ca^2+^ was reintroduced to the cardiomyocytes by stepwise pelleting cells at 20*g* and resuspended in buffers with increasing concentrations of CaCl_2_, with a final Ca^2+^ concentration of 900 *µ*M. The cells were seeded on laminin-coated plates (10 *µ*g/well) (Thermo Fisher Scientific) in plating medium (MEM with HBSS [Lonza], 10% calf serum, 10 mM BDM [Sigma-Aldrich], 100 U/ml penicillin [HyClone], and 2 mM L-glutamine [HyClone]), which had been equilibrated at 37°C, 18% O_2_, and 2% CO_2_ to reach an optimal pH.

### Gene expression analysis

Total RNA was extracted from snap-frozen mouse heart tissue using RNeasy Fibrous Tissue Kit (QIAGEN) or isolated from cultured mouse cardiomyocytes using the RNeasy mini kit (QIAGEN). cDNA was synthesized using High-Capacity cDNA Reverse Transcription Kit (Applied Biosystems) with random primers. Quantitative real-time PCR amplification of cDNA was performed using the SsoAdvanced Universal SYBR Green (Bio-Rad) or TaqMan Fast Advanced Master Mix (Thermo Fisher Scientific). mRNA expression was normalized to HPRT mRNA expression and expressed as fold change compared with control average using the ΔΔCT method. Sequences of primers and TaqMan references used for real-time PCR are listed in Table S3.

Table S3. Sequences of primers or TaqMan assay references used for gene expression analysis.

### Western blot analysis

Equal amounts of total protein were loaded and run on a NuPAGE 4–12% Bis-Tris gel (Invitrogen). Blots were probed with the following antibodies (used at 1:1,000): anti-Plin5 (GP31) from PROGEN; anti-Akt p-Ser473 (4060), anti-Akt (9272), anti-p70 S6K p-Thr389 (9206), anti-p70 S6K (2708), anti-E4-BP1 p-Thr37/46 (9459), anti-sarco/endoplasmic reticulum calcium ATPase (SERCA)2 (9580), anti-PLN p-Ser16/Thr17 (8496), anti-PLN (14562), anti-p38 p-Thr180/182 (4511), anti-p38 (9212), anti-Erk1/2 p-Thr202/Tyr204 (4376), anti-Erk1/2 (4695), anti-p110 α (4249), and anti-p85 (4292) from Cell Signaling Technology; anti-FLAG from Sigma-Aldrich; anti-HPRT (109021), anti-p-α 1 Na/K ATPase (7671), anti-β-actin (8226), and anti-calreticulin (92516) from Abcam; and anti-PLN p-Ser16 (A010-12AP) and anti-PLN p-Thr17 (A010-12AP) from Badrilla. In the analysis of the MAPK (Erk1/2, p38) and Akt/mTOR signaling pathways, the specific phosphorylation sites for each kinase were chosen because these sites have been shown to be critical for the kinase function. HRP-coupled secondary antibodies (used at 1:3,000) were from Dako. Immunoblots were visualized with Immobilon Western Chemiluminescent Horseradish Peroxidase Substrate (Millipore) and detected with a ChemiDoc Touch (Bio-Rad) camera. Bands were quantified with Image Lab 5.2.1 (Bio-Rad). Coomassie staining was done with the Novex Colloidal Blue Staining Kit (LC6025; Invitrogen) according to the manufacturer’s instructions.

### Co-immunoprecipitation

Cardiomyocytes were mixed in buffer, pH 7.4, containing 10  mM Hepes, 150  mM NaCl, 1  mM EDTA, 0.5 mM EGTA, 1% Triton X-100, 0.1 mM PMSF, protease, and phosphatase inhibitors (Roche) for 2 h at 4°C, and the homogenates were centrifuged at 10,000*g* for 10 min at 4°C. For FLAG immunoprecipitation, the supernatant was collected and 500 *µ*g of total protein was bound to 1.5 mg of Pierce Anti-FLAG Magnetic Agarose (A36797) overnight at 4°C. Beads were washed four times with 50 mM TEAB in ultrapure water. The bait–prey complex was eluted twice using 1% formic acid in ultrapure water in a ThermoMixer device (5382000015 from Eppendorf) at 25°C/1,400 rpm for 5 min. Elution fractions were lyophilized and analyzed using SDS–PAGE, Western blots, and mass spectrometry. For Plin5 immunoprecipitation, 500 *µ*g of total protein was bound to Dynabeads Protein A for Immunoprecipitation (10002D) precoupled with 4 μg of perilipin 5 polyclonal antibody (26951-1-AP) or 4 *µ*g of rabbit non-immune IgG (NB810-56910) overnight at 4°C. Beads were washed four times with buffer, pH 7.4, containing 10 mM Hepes, 150  mM NaCl, 1  mM EDTA, 0.5 mM EGTA, 1% Triton X-100, 0.1 mM PMSF, protease, and phosphatase inhibitors (Roche). The bait–prey complex was eluted twice using 2X Laemmli sample buffer (1610747; Bio-Rad) in a ThermoMixer device at 50°C/1,400 rpm for 10 min. After adding a final 50 mM concentration of DTT, the elution fractions were analyzed using SDS–PAGE and Western blots.

### Proteomic analysis

Lyophilized elution fractions from the co-immunoprecipitation were reduced with DL-dithiothreitol (DTT, 100 mM) at 60°C for 30 min and then processed according to the filter-aided sample preparation method modified from Wiśniewski et al (2009). In short, reduced samples were transferred onto Microcon-30kD centrifugal filters (Merck) washed repeatedly with 50 mM triethylammonium bicarbonate (TEAB) and once with digestion buffer (0.5% sodium deoxycholate [SDC] and 50 mM TEAB). The reduced cysteine side chains were alkylated with 10 mM methyl methanethiosulfonate (MMTS) in digestion buffer for 30 min at room temperature, and the samples were then repeatedly washed with digestion buffer. Samples were digested with trypsin (0.3 *µ*g; Pierce MS-grade Trypsin; Thermo Fisher Scientific) at 37°C overnight, and an additional portion of trypsin (0.3 *µ*g) was added and incubated for another 3 h. The peptides were collected by centrifugation, and isobaric labeling was performed using tandem mass tag (TMT10plex) reagents (Thermo Fisher Scientific) according to the manufacturer’s instructions. The labeled samples were combined into one pooled sample, acetonitrile was evaporated using vacuum centrifugation, and the sample was purified using High Protein and Peptide Recovery Detergent Removal Spin Column (Thermo Fisher Scientific) according to the manufacturer’s instructions. SDC was removed by acidification with 10% TFA and subsequent centrifugation. The supernatants were purified using Pierce peptide desalting spin columns (Thermo Fisher Scientific) according to the manufacturer’s instructions. The purified peptide samples were dried on Speedvac and reconstituted in 3% acetonitrile, 0.2% formic acid for the LC–MS/MS analysis.

Samples were analyzed on an Orbitrap Fusion Tribrid spectrometer interfaced with an Easy-nLC 1200 nanoflow liquid chromatography system (both from Thermo Fisher Scientific). Peptides were trapped on an Acclaim PepMap 100 C18 trap column (100 μm × 2 cm, particle size 5 μm; Thermo Fisher Scientific) and separated on an in-house packed analytical column (75 μm × 35 cm, particle size 3 μm, Reprosil-Pur C18, Dr. Maisch) from 5% to 12% B over 5 min, 12–35% B over 70 min followed by an increase to 100% B for 5 min, and 100% B for 10 min at a flow of 300 nl/min. Solvent A was 0.2% formic acid, and solvent B was 80% acetonitrile, 0.2% formic acid. MS scans were performed at 120,000 resolution, m/z range 375–1,375. MS/MS analysis was performed in a data-dependent mode, with top speed cycle of 3 s for the most intense doubly or multiply charged precursor ions. Precursor ions were isolated in the quadrupole with a 0.7-m/z isolation window, with dynamic exclusion set to 10 ppm and duration of 60 s. Isolated precursor ions were subjected to collision-induced dissociation at 35 collision energy with a maximum injection time of 50 ms. Produced MS2 fragment ions were detected in the ion trap followed by multinotch (simultaneous) isolation of the top 10 most abundant fragment ions for further fragmentation (MS3) by higher energy collision dissociation at 65% and detection in the Orbitrap at 50,000 resolutions, m/z range 100–500.

Data were analyzed using Proteome Discoverer version 2.4 (Thermo Fisher Scientific). The raw file was matched against the SwissProt mouse database (Mars 2020) using Mascot 2.5.1 (Matrix Science) as a database search engine with peptide tolerance of 5 ppm and fragment ion tolerance of 0.6 D. Tryptic peptides were accepted with zero missed cleavage, mono-oxidation on methionine was set as a variable modification, and methylthiolation on cysteine and TMT-6 reagent modification on lysine and peptide N-terminus were set as a fixed modification. Percolator was used for PSM validation with the strict FDR threshold of 1%. Reporter ion intensities were quantified in MS3 spectra at 0.003 D mass tolerance using the S/N values as abundance and normalized on the total protein abundance within the Proteome Discoverer 2.4 workflow. Only the values for the unique peptides were used for quantitation.

### In situ proximity ligation assay

**Figure S7.**
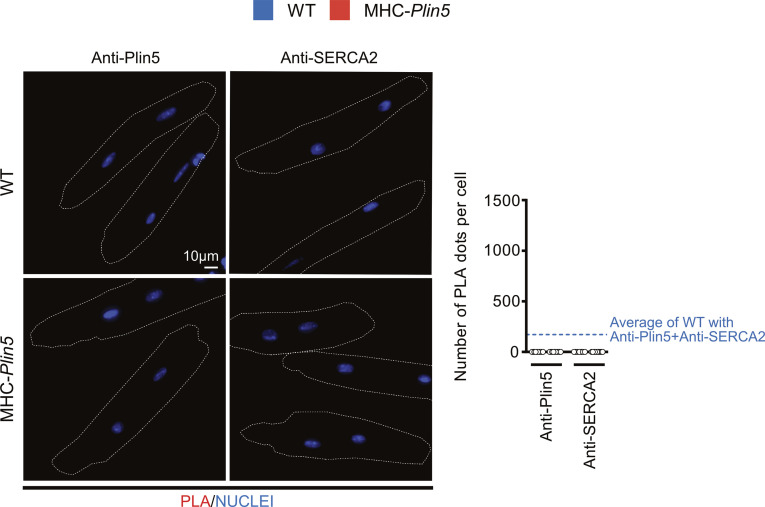
Technical negative controls for in situ proximity ligation assay. Left, representative images of in situ interactions between Plin5 and SERCA2 using PLA in adult WT and MHC-*Plin5* cardiomyocytes. Nuclei were stained using DRAQ5. PLA signal is red, and DRAQ5 signal is blue. Right, quantification of the PLA fluorescent dots (n_mice_ = 2 [WT], 2 [MHC-*Plin5*]; n_cell_ = 10 [WT-anti-Plin5], 10 [MHC-*Plin5*-anti-Plin5], 7 [WT-anti-SERCA2], 11 [MHC-*Plin5*-anti-SERCA2]). Values are the mean ± SEM.

Primary cardiomyocytes were fixed in 4% paraformaldehyde for 15 min. After permeabilization in 0.1% Triton X-100 in PBS for 10 min and blocking, the cells were probed with perilipin 5 polyclonal antibody (26951-1-AP from ProteinTech) and SERCA2 ATPase monoclonal antibody (MA3-910 from Invitrogen) in combination or alone (used as technical negative controls and presented in Fig S7). Non-immune rabbit IgG (NB810-56910 from Novus) and mouse IgG1 (14-4714-85 from Invitrogen) were used as the biological negative control. For in situ proximity ligation assay, all incubations were performed in a humidity chamber and according to Navinci’s recommendations using NaveniFlex MR Kit (Navinci (formerly Olink Bioscience)). A nucleus staining solution with DRAQ5 (final 5 *µ*M, 62251; Thermo Fisher Scientific) was used instead of DAPI for 5 min at RT. Coverslips were mounted with ProLongTM Glass Antifade Mountant (P36982; Invitrogen), and fluorescence was visualized with an ECLIPSE Ni-E fluorescence microscope (Nikon) using a 40× Plan Fluor (NA 1.30) objective. Pictures were obtained using a Zyla 5.5 sCMOS camera with a fixed time exposure for all conditions. For analysis, z-scan images covering the whole cardiomyocyte thickness were deconvoluted using NIS-Elements Imaging Software (Nikon). Numbers of proximity ligation assay dots per cell were then counted on 2D maximum intensity projection along the z-axis. All image processing steps were carried out in ImageJ.

### Blue-native and SDS–PAGE 2D separation

Cardiomyocytes were resuspended in buffer, pH 7.4, containing 10 mM Hepes, 150 mM NaCl, 1 mM EDTA, 0.5 mM EGTA, 1% Triton X-100, 0.1 mM PMSF, and protease and phosphatase inhibitors (Roche) for 2 h at 4°C, and the homogenates were centrifuged at 10,000*g* for 10 min at 4°C. Blue-native PAGE and two-dimensional PAGE were performed according to the user guide of Novex native gel electrophoresis system (Life Technologies). Briefly, for first-dimension blue-native PAGE, 30 μg of proteins combined with NativePAGE 5% G-250 Sample Additive (BN2004 from Invitrogen) was loaded onto a NativePAGE 3–12% Bis-Tris gel (BN2011BX10 from Invitrogen). Gels were either kept for Coomassie staining, transferred to a PVDF membrane for immunoblotting, or used for second-dimension separation. For second-dimension separation, sample gel lines were carefully excised and reduced with 1× NuPAGE reducing agent in 1× NuPAGE LDS sample buffer (Invitrogen) for 15 min at RT. Then, cysteine alkylation was done with iodoacetamide for 15 min at RT. Gel strip was immediately applied to the second dimension on a NuPAGE Novex 4–12% Bis-Tris ZOOM Gel (NP0330BOX from Invitrogen), separated, and either used for Coomassie staining or transferred to a PVDF membrane for immunoblotting.

### Ca^2+^ imaging

Intracellular free Ca^2+^ was recorded in isolated mouse cardiomyocytes using the organic Ca^2+^ indicator Fluo-4-AM (Molecular Probes). Time-lapse recordings were performed on a custom-made microscope based around an inverted Nikon Eclipse TI2 body equipped with a 40 × 1.15 NA water immersion objective (MRD77410; Nikon). A 488-nm laser (06-MLD; Cobolt) served as the excitation light source for Fluo-4. An EGFP filter set (excitation, dichroic, and emission) (F36-525; AHF analysentechnik) mounted in a filter cube (F91-963; AHF analysentechnik) was used to separate excitation light from emission light. The emission light was recorded on an EMCCD camera (iXon 897 Ultra; Andor).

Cardiomyocytes isolated from either WT or MHC-*Plin5* mice were plated on confocal glass-bottomed 35-mm dishes coated with laminin for 2 h in plating medium and then loaded with 5 μM Fluo-4-AM for 20 min at room temperature in modified Tyrode’s solution (140 mM NaCl, 3.6 mM KCl, 0.5 mM MgSO_4_, 0.5 mM NaH_2_PO_4_, 2 mM NaHCO_3_, 5 mM Hepes, 5 mM glucose, and 2.6 mM CaCl_2_). Cells were paced at 0.4 ms, 35 V, and 0.5 Hz using a pair of platinum wires placed on the opposite sides of the dish chamber and connected to a MyoPacer Field Stimulator (IonOptix). After a 5-min period of stabilization, Ca^2+^ transients (sparks) were recorded in a 3-min acquisition. Ca^2+^ transients of excitable, rod-shaped cardiomyocytes were eligible for analysis when lacking automaticity and showing regular intracellular Ca^2+^ amplitude clearance under field stimulation. For isoproterenol-stimulated recordings, modified Tyrode’s solution was replaced by modified Tyrode’s solution containing 10 nM isoproterenol. The first 5 min of isoproterenol stimulation allowed preselection of excitable, rod-shaped cardiomyocytes. Pacing was then turned on for a 5-min period of stabilization and followed by additional 10-min acquisition. To measure SR Ca^2+^ content, both baseline and isoproterenol-stimulated recordings were ended by a 1-min stop pacing period before 10 mM caffeine was added. The software Micro-Manager was used to control the hardware of the microscope and to sequentially acquire and store images at 97 frames/s during time-lapse imaging.

Data were processed using the Fiji (https://fiji.sc) software package and a custom-written plugin. The source code for the plugin is available upon request. Background was subtracted from each frame of a time-lapse recording before calculating ΔF/F₀. The background was estimated by averaging the pixel values from a region that did not contain any cells or bright spots (such as from reflections of debris on the coverslip). A region of interest was then drawn around the outlines of a cell, and the averaged pixel values of this region of interest were plotted over time using built-in plot functions of Fiji.

To normalize the differences in dye concentration and cell thickness between cells/recordings, ΔF/F₀ was calculated from the values in the plot. F₀ was estimated by averaging the 10–40 frames that precede Ca^2+^ rise.

From the resulting ΔF/F₀ traces, the spike amplitude and rate of Ca^2+^ rise were calculated for a minimum of 30 spikes from each cell. The amplitude was calculated by finding maxima in the data using the “MaximumFinder” class of Fiji. The rate of rise was calculated by dividing amplitude by time to peak. One randomly chosen spike from each cell was fit to a single exponential decay to calculate systolic decay rate. For caffeine analysis, the final electrically stimulated and caffeine-induced Ca^2+^ transients were fit to a single exponential decay to calculate systolic and caffeine-induced decay rates, respectively.

### Statistical analyses

Statistical analysis and graphics were performed using GraphPad Prism 9.3.1 (GraphPad Software). Details of the statistical analysis, including numbers of mice, are indicated in the figure legends. Comparison between two groups was performed by unpaired two-tailed *t* test, whereas comparison between multiple groups was performed by two-way ANOVA followed by Sidak’s multiple comparisons post hoc tests. The significance level was set at *P* < 0.05.

## Data Availability

The mass spectrometry proteomics data have been deposited to the ProteomeXchange Consortium via the PRIDE (Perez-Riverol et al, 2022) partner repository with the dataset identifier PXD035121.

## Supplementary Material

Reviewer comments
