# Supplementary material for: Cardiac Plin5 interacts with SERCA2 and promotes calcium handling and cardiomyocyte contractility
Source: Life Sci Alliance. 2023 Jan 30;6(4):e202201690. doi: 10.26508/lsa.202201690 (PMC9887753; doi:10.26508/lsa.202201690)

Figure 3B

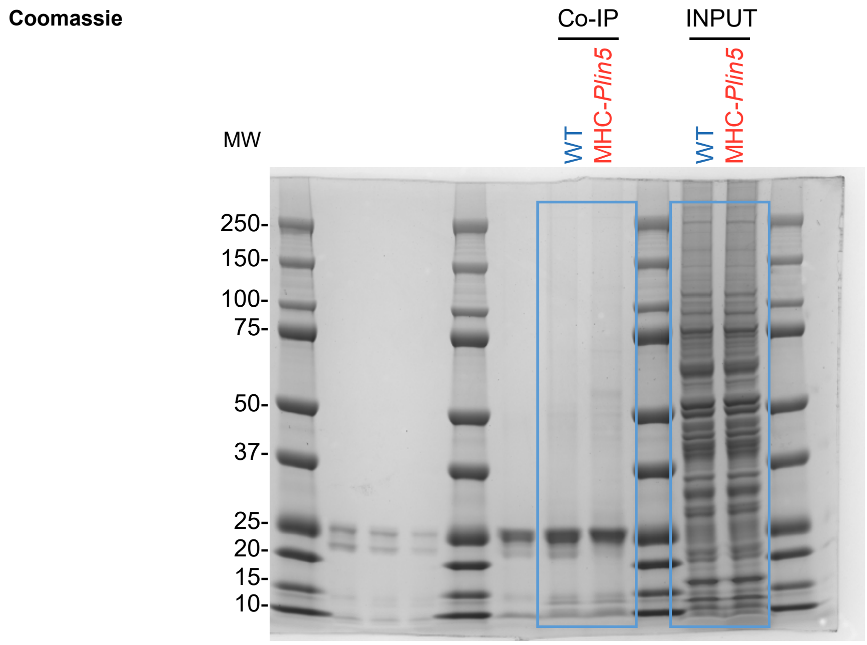
Coomassie
Co-IP
INPUT
MW
WT
MHC-Plin5
WT
MHC-Plin5
250-
150-
100-
75-
50-
37-
25-
20-
15-
10-

Figure 3C

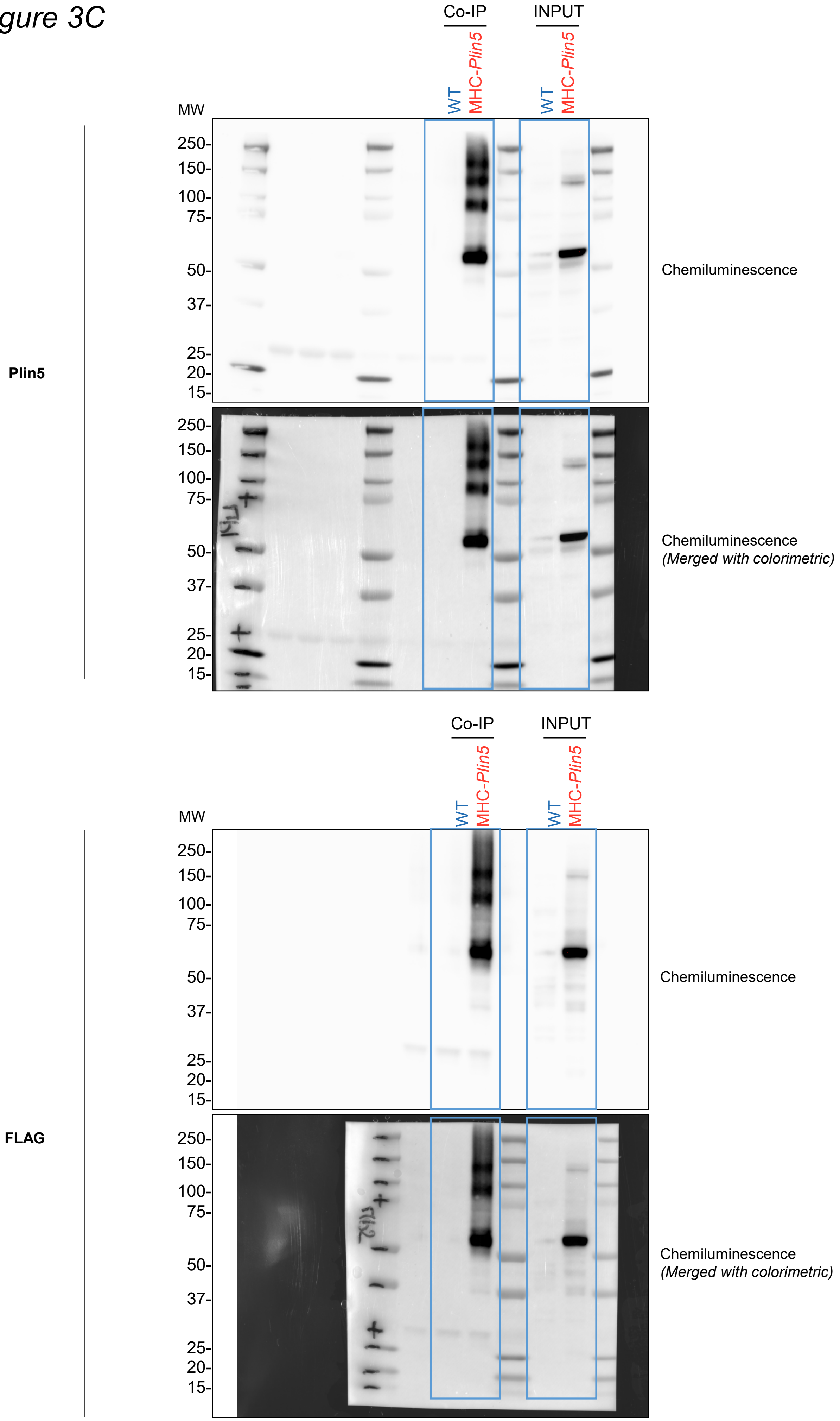
Co-IP
INPUT
WT
MHC-Plin5
WT
MHC-Plin5
MW
250-
150-
100-
75-
50-
37-
25-
20-
15-
Plin5
Chemiluminescence
Chemiluminescence
(Merged with colorimetric)
Co-IP
INPUT
WT
MHC-Plin5
WT
MHC-Plin5
MW
FLAG
Chemiluminescence
Chemiluminescence
(Merged with colorimetric)

Figure 4A

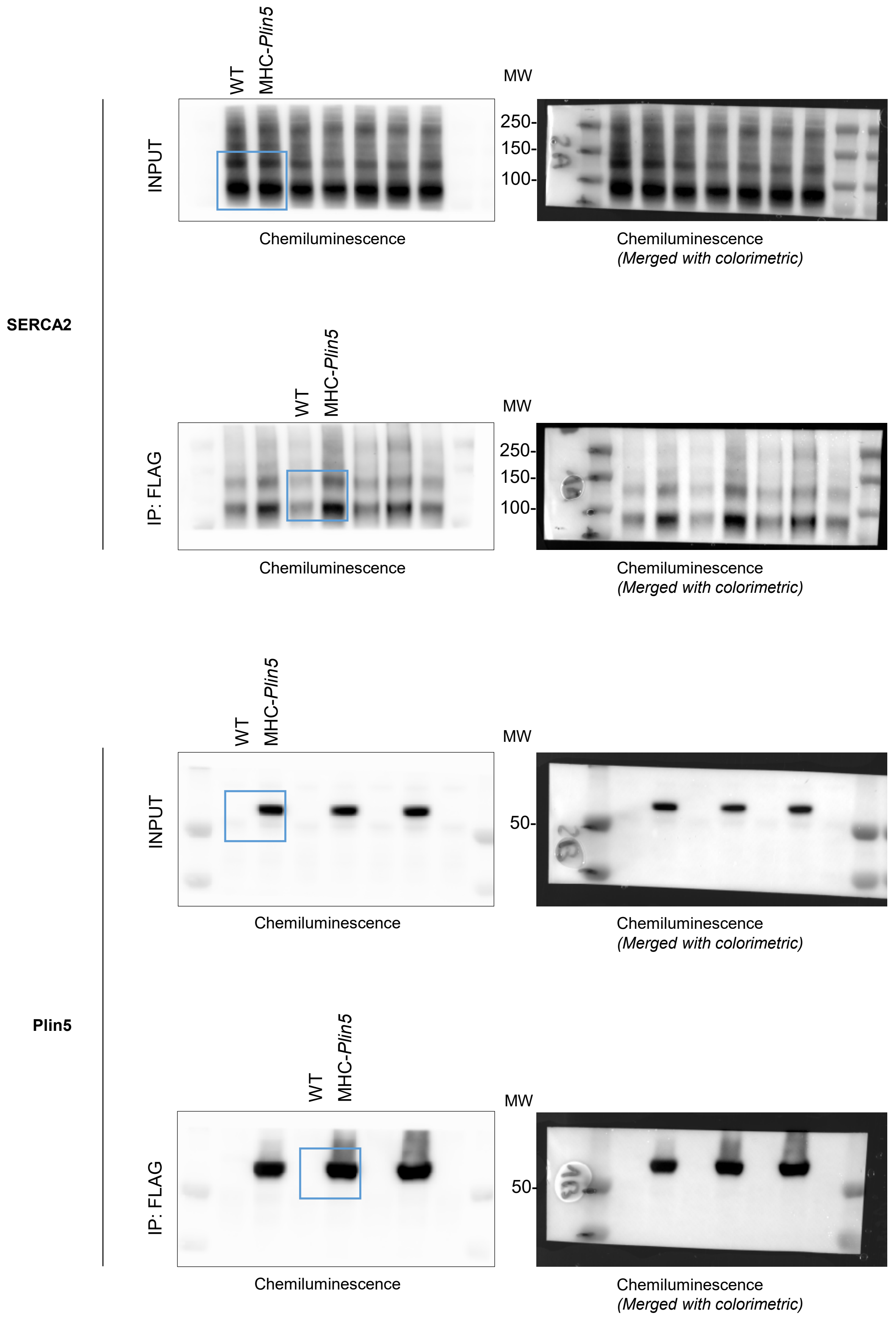

WT
MHC-*Plin5*
MW
INPUT
250-
150-
100-
Chemiluminescence
Chemiluminescence
(Merged with colorimetric)
SERCA2
WT
MHC-*Plin5*
MW
IP: FLAG
250-
150-
100-
Chemiluminescence
Chemiluminescence
(Merged with colorimetric)
WT
MHC-*Plin5*
MW
INPUT
50-
Chemiluminescence
Chemiluminescence
(Merged with colorimetric)
Plin5
WT
MHC-*Plin5*
MW
IP: FLAG
50-
Chemiluminescence
Chemiluminescence
(Merged with colorimetric)

Figure 4B

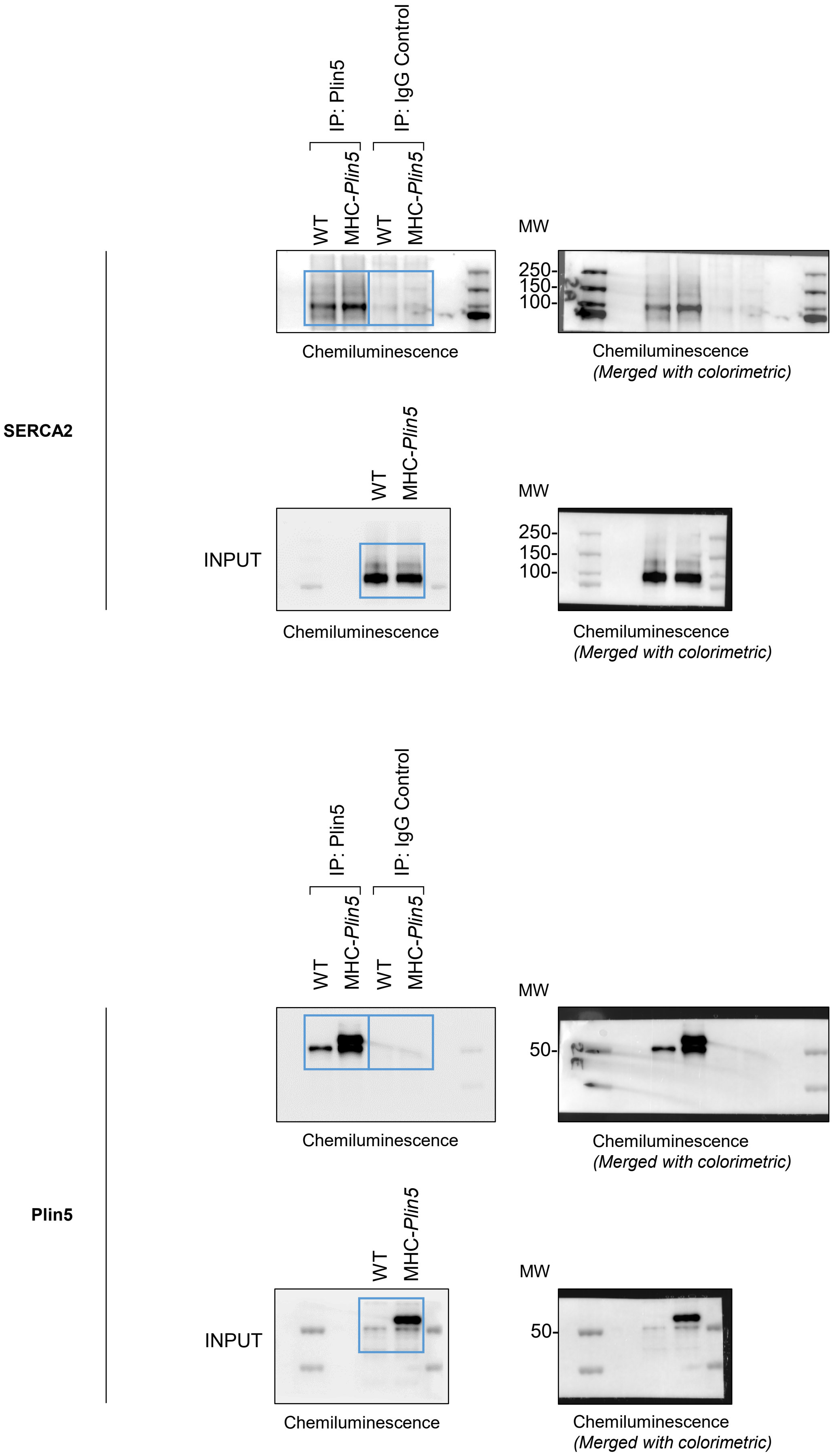

IP: Plin5
IP: IgG Control
WT
MHC-*Plin5*
WT
MHC-*Plin5*
MW
250-
150-
100-
Chemiluminescence
Chemiluminescence
(Merged with colorimetric)
SERCA2
WT
MHC-*Plin5*
INPUT
MW
250-
150-
100-
Chemiluminescence
Chemiluminescence
(Merged with colorimetric)
IP: Plin5
IP: IgG Control
WT
MHC-*Plin5*
WT
MHC-*Plin5*
MW
50-
Chemiluminescence
Chemiluminescence
(Merged with colorimetric)
Plin5
WT
MHC-*Plin5*
INPUT
MW
50-
Chemiluminescence
Chemiluminescence
(Merged with colorimetric)

Figure 5B-Part1

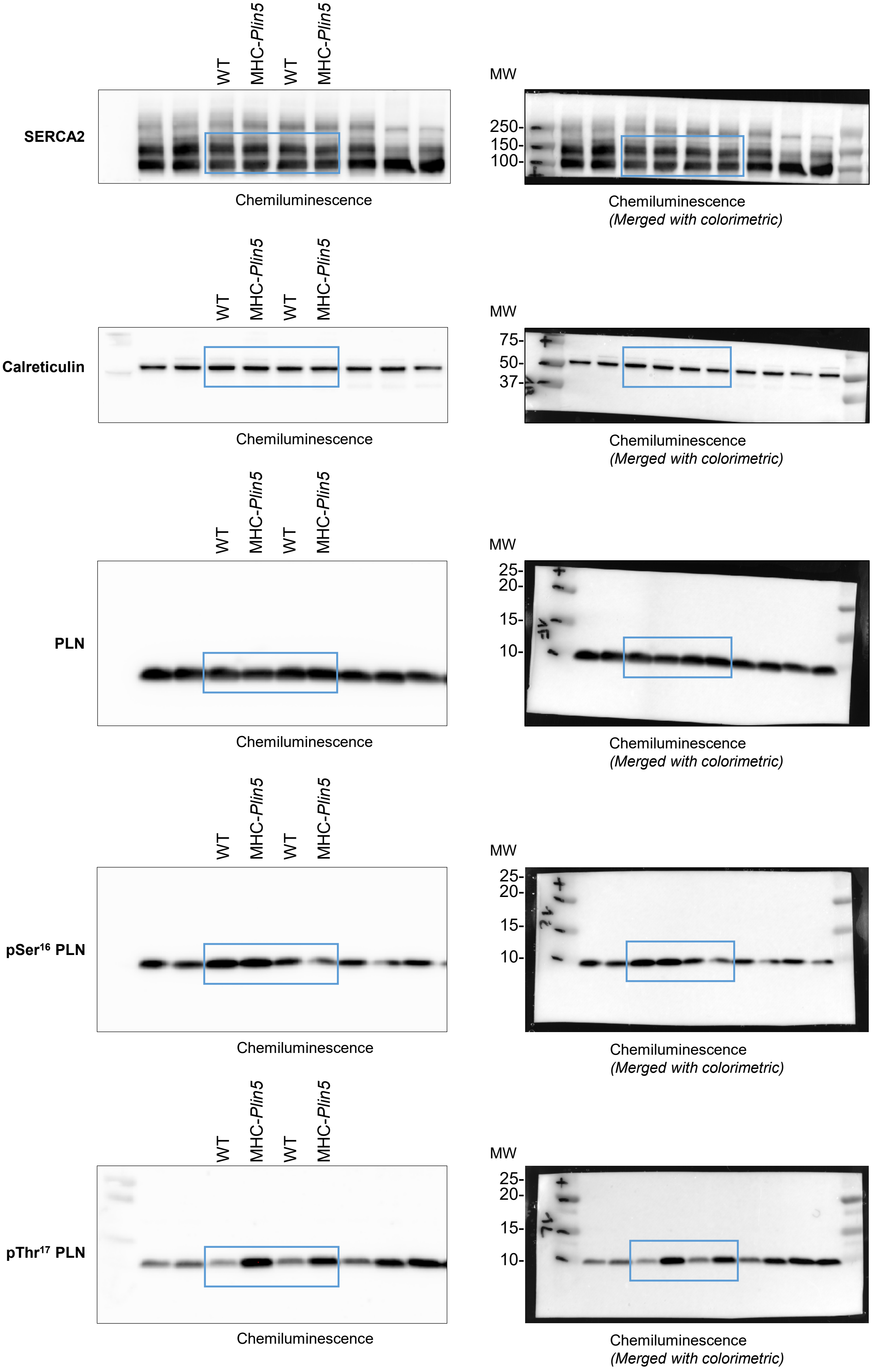

Figure 5B-Part2

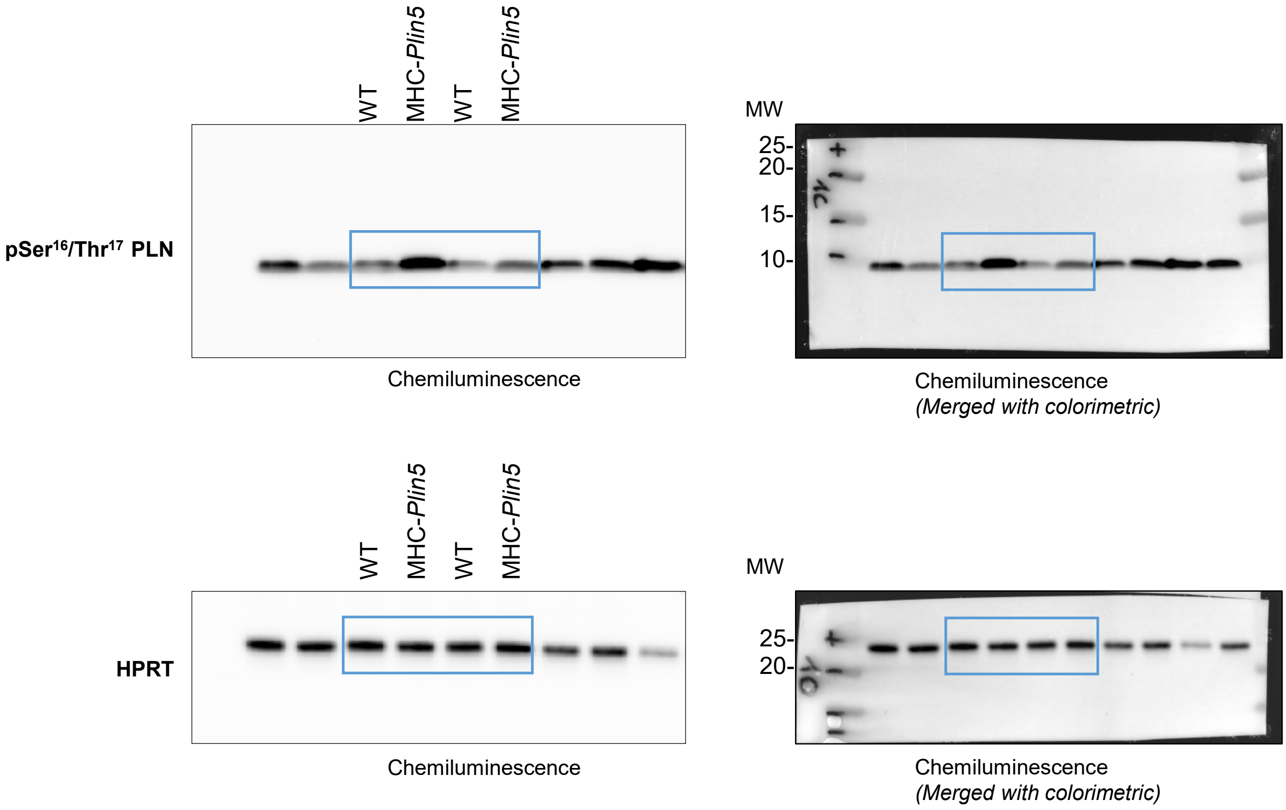

Figure S1B

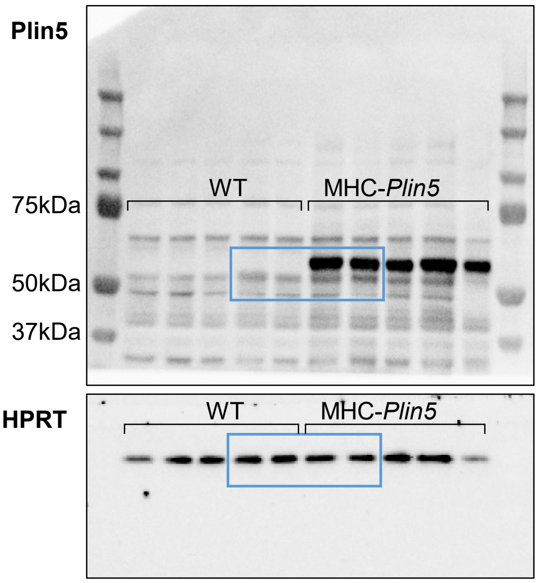

Figure S1D

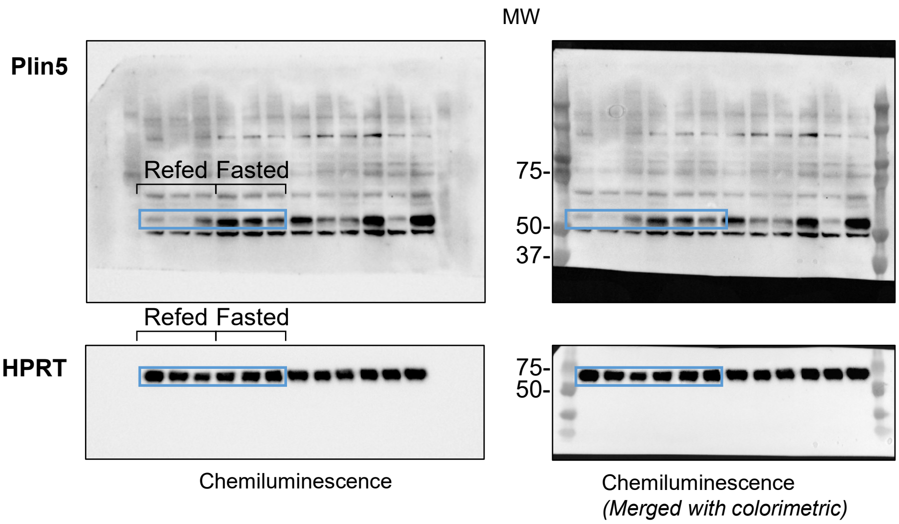

Figure S2A

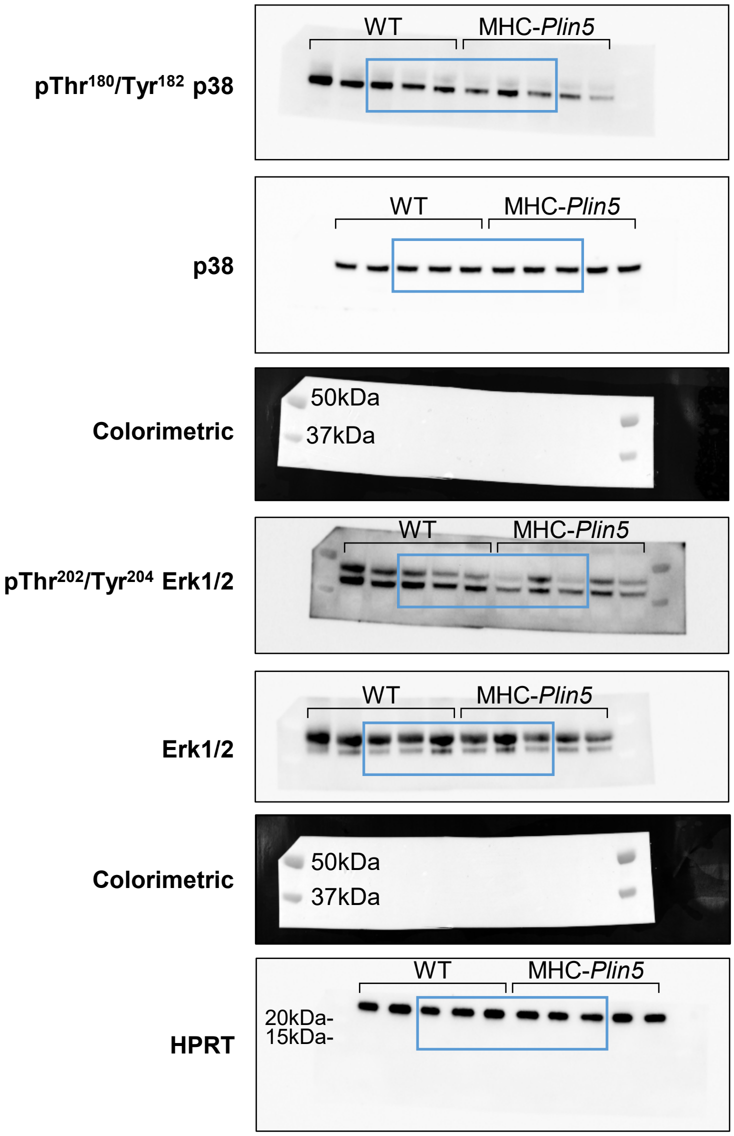

pThr180/Tyr182 p38
WT
MHC-Plin5
p38
WT
MHC-Plin5
Colorimetric
50kDa
37kDa
pThr202/Tyr204 Erk1/2
WT
MHC-Plin5
Erk1/2
WT
MHC-Plin5
Colorimetric
50kDa
37kDa
HPRT
WT
MHC-Plin5
20kDa-
15kDa-

Figure S2C

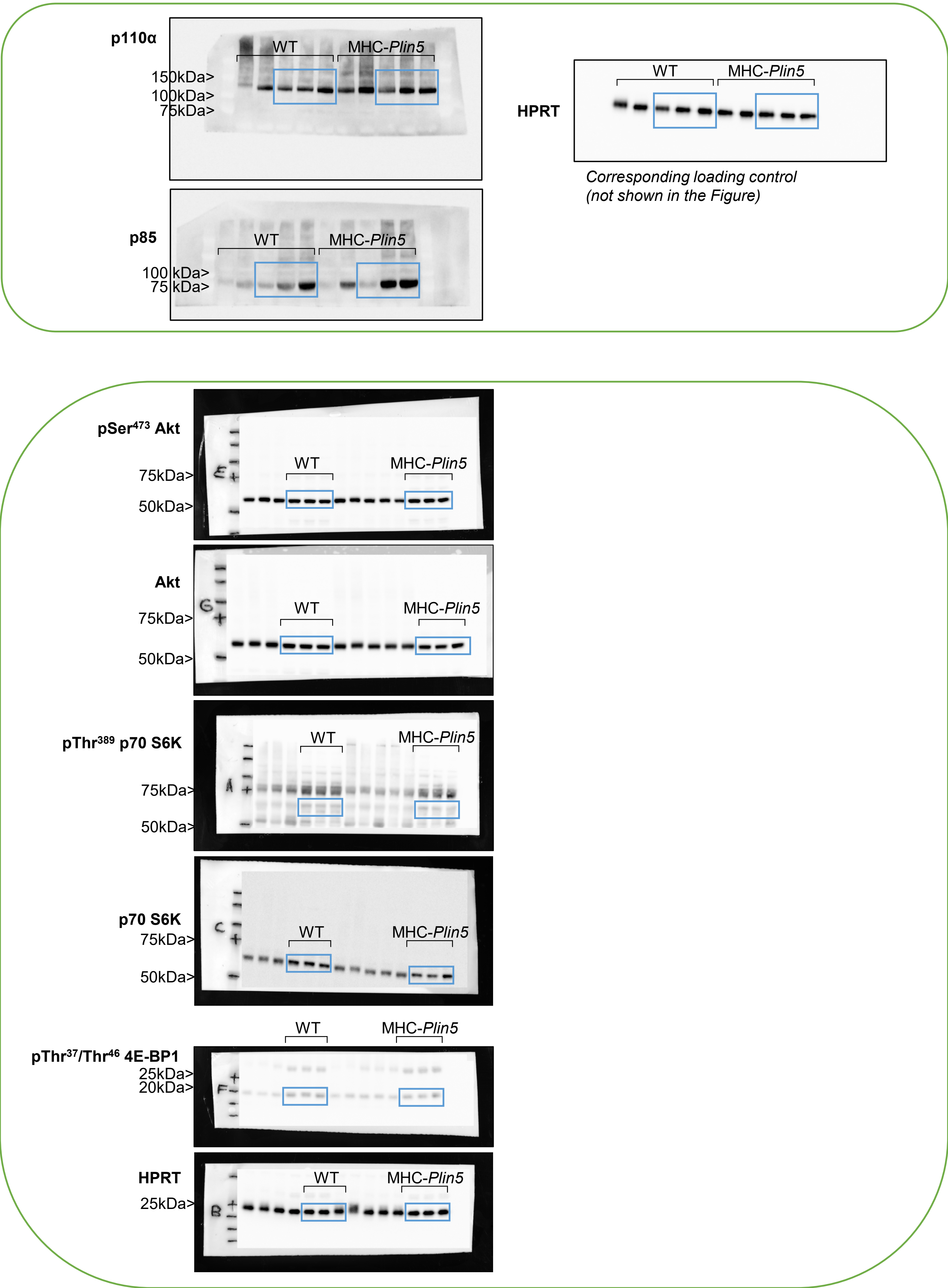
p110α
WT
MHC-*Plin5*
150kDa>
100kDa>
75kDa>
HPRT
WT
MHC-*Plin5*
Corresponding loading control (not shown in the Figure)
p85
WT
MHC-*Plin5*
100 kDa>
75 kDa>
pSer473 Akt
75kDa>
50kDa>
WT
MHC-*Plin5*
Akt
75kDa>
50kDa>
WT
MHC-*Plin5*
pThr389 p70 S6K
75kDa>
50kDa>
WT
MHC-*Plin5*
p70 S6K
75kDa>
50kDa>
WT
MHC-*Plin5*
pThr37/Thr46 4E-BP1
25kDa>
20kDa>
WT
MHC-*Plin5*
HPRT
25kDa>
WT
MHC-*Plin5*

Figure S4A

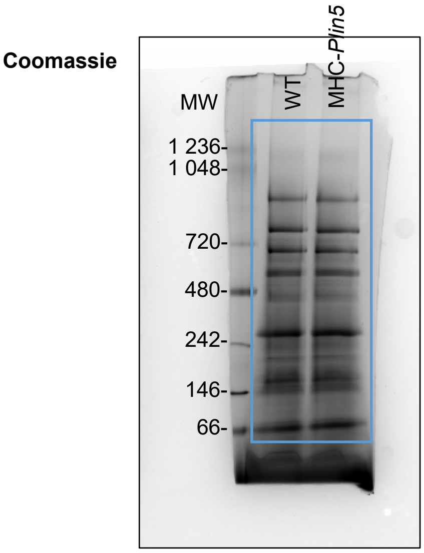

Figure S4B

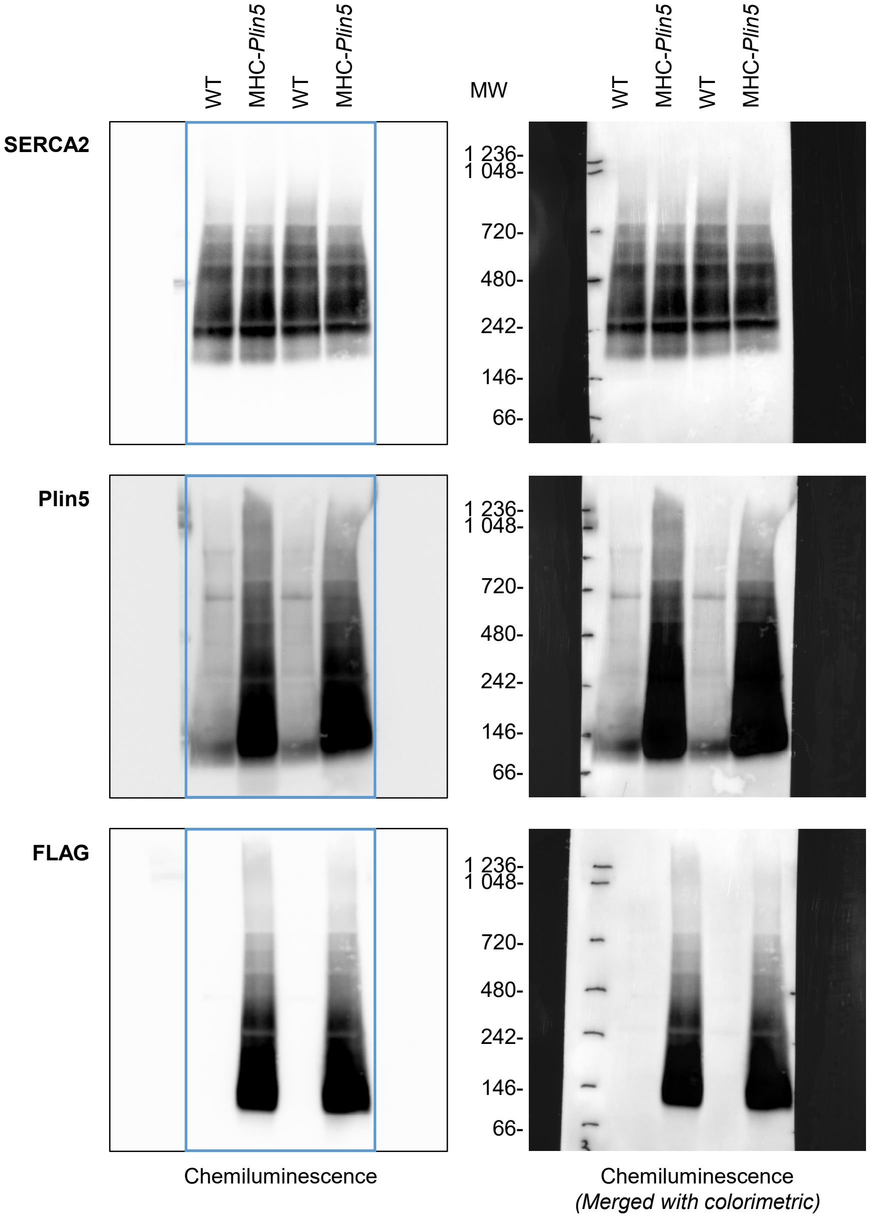

Figure S4C

(Horizontal 1D BN PAGE: see Figure S4A)

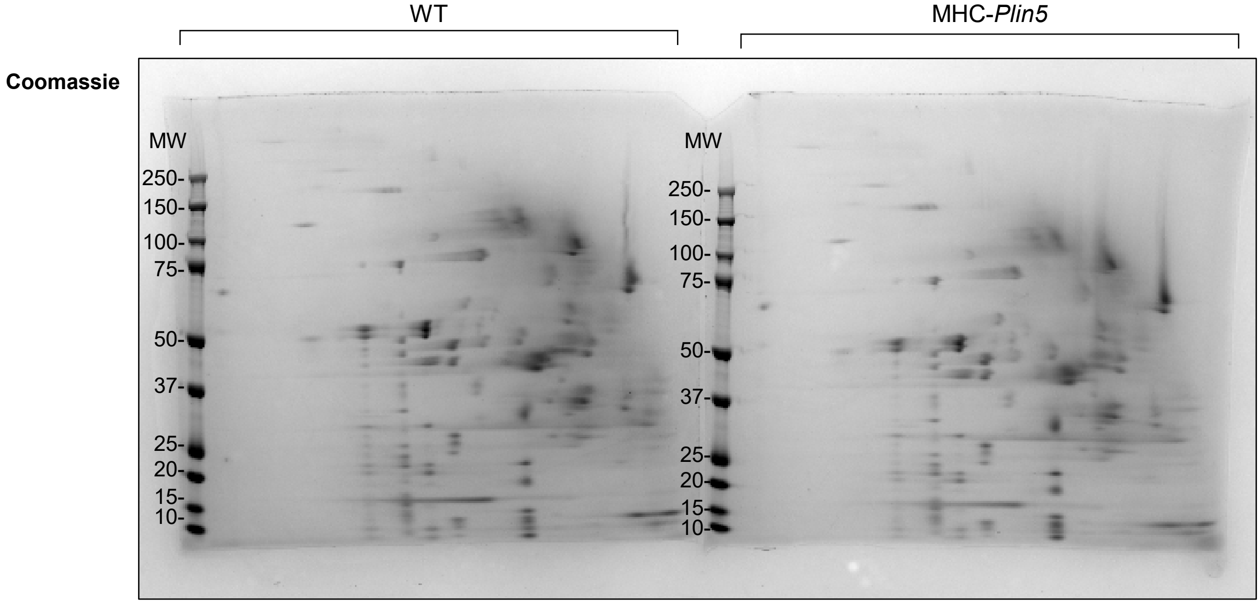

Figure S4D

(Horizontal 1D BN PAGE: see Figure S4B)

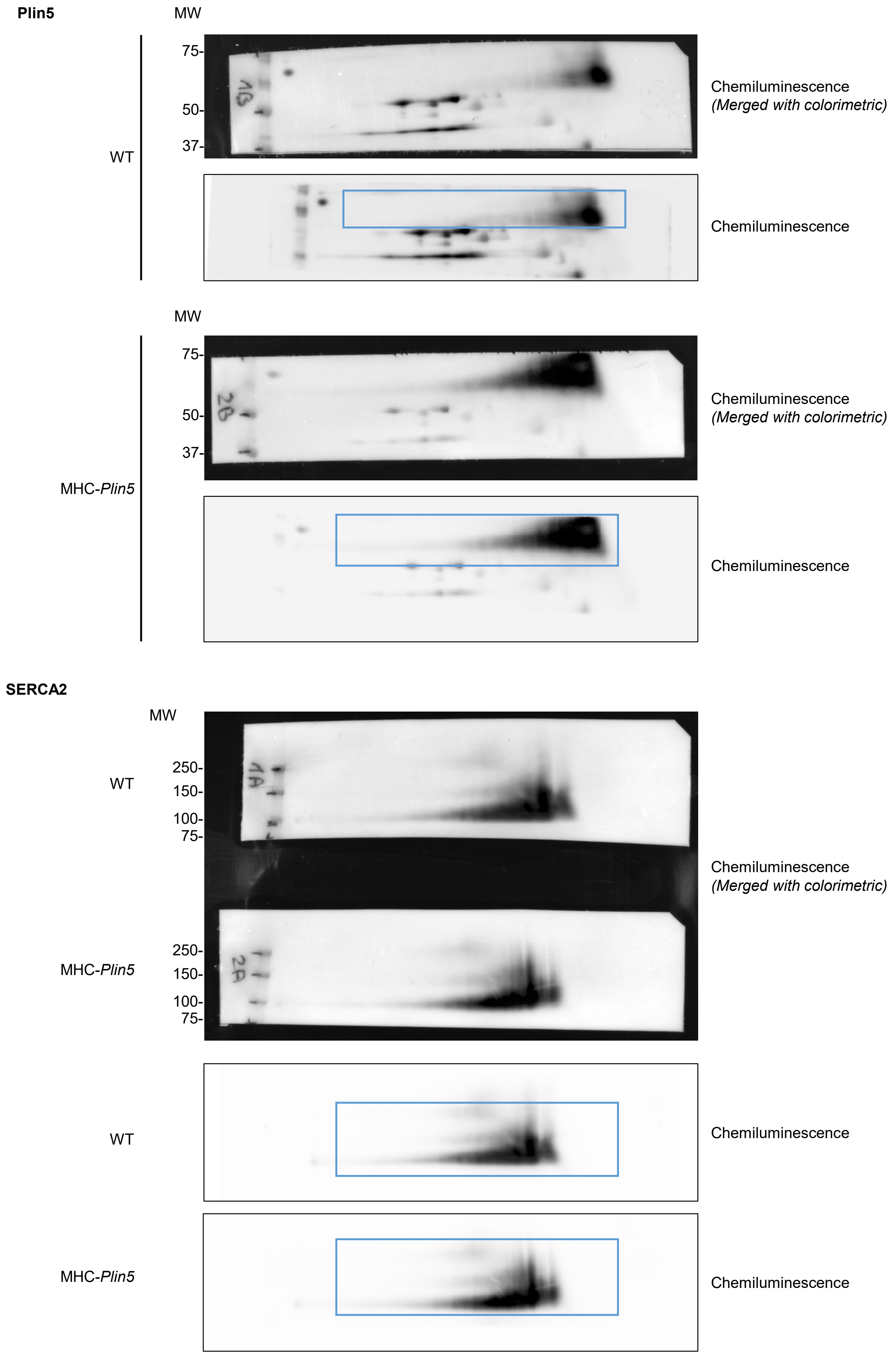

Supplement: Supplementary file 2 [file LSA-2022-01690_SdataF3_F4_F5_FS1_FS2_FS4.pdf]
