# Supplementary material for: Cardiac Plin5 interacts with SERCA2 and promotes calcium handling and cardiomyocyte contractility
Source: Life Sci Alliance. 2023 Jan 30;6(4):e202201690. doi: 10.26508/lsa.202201690 (PMC9887753; doi:10.26508/lsa.202201690)
Supplement: Supplementary file 3 [file LSA-2022-01690_TableS1.docx]

**Table S1. Metabolic and plasma parameters in WT and MHC-Plin5 mice.**

| **Parameters** | **WT (n=6-10)** | **MHC-*Plin5* (n=6-9)** | **p value** |
| --- | --- | --- | --- |
| Body weight (g) | 26.2 ± 0.5 | 25.9 ± 0.6 | 0.702 |
| Insulin (ng/ml) | 1.94 ± 0.13 | 2.03 ± 0.15 | 0.647 |
| Glucose (mM) | 9.60 ± 0.57 | 9.10 ± 0.53 | 0.529 |
| Cholesterol (mM) | 3.35 ± 0.21 | 3.50 ± 0.14 | 0.580 |
| Triglycerides (mM) | 0.76 ± 0.06 | 0.85 ± 0.03 | 0.202 |

Plasma from WT and MHC-Plin5 taken 4 h after fasting. Data are presented as mean ± SEM; p values calculated by Student’s t test.
