# Supplementary material for: Cardiac Plin5 interacts with SERCA2 and promotes calcium handling and cardiomyocyte contractility
Source: Life Sci Alliance. 2023 Jan 30;6(4):e202201690. doi: 10.26508/lsa.202201690 (PMC9887753; doi:10.26508/lsa.202201690)
Supplement: Supplementary file 4 [file LSA-2022-01690_TableS2.docx]

**Table S2. Heart, Liver and Lung weight in both young and naturally aging WT and MHC-Plin5 mice.**

|  | **11-week-old** | | **22/27-week-old** | |
| --- | --- | --- | --- | --- |
| **Parameters** | **WT**  **(n=9-12)** | **MHC-*Plin5***  **(n=8-15)** | **WT**  **(n=9-17)** | **MHC-*Plin5***  **(n=7-16)** |
| Body weight (g) | 28.3 ± 0.5 | 27.7 ± 0.5 | 36.7 ± 1.4  ***p<0.0001 | 35.0 ± 1.2  ***p<0.0001 |
| Heart Weight (mg) | 145.2 ± 5.2 | 159.4 ± 3.5  #p=0.0295 | 157.6 ± 3.5 | 173.2 ± 3.2  *p=0.0217, ##p=0.0073 |
| Lungs Weight (mg) | 158.1 ± 5.6 | 165.9 ± 1.7 | 177 ± 3.2  **p=0.0033 | 181.6 ± 4.7  *p=0.0131 |
| Liver Weight (mg) | 1350.7 ± 31.8 | 1313.7 ± 32.4 | 1772.1 ± 40.1  ***p<0.0001 | 1671.8 ± 74.0  ***p<0.0001 |

Weights of the indicated organs in 11-week-old and 22 to 27-week-old WT and MHC-Plin5 mice. Data are presented as mean ± SEM; p values calculated Two-way ANOVA followed by a Sidak's multiple comparisons post hoc test. # vs respective WT and * vs respective 11-week-old mice.
