# Supplementary material for: Cardiac Plin5 interacts with SERCA2 and promotes calcium handling and cardiomyocyte contractility
Source: Life Sci Alliance. 2023 Jan 30;6(4):e202201690. doi: 10.26508/lsa.202201690 (PMC9887753; doi:10.26508/lsa.202201690)
Supplement: Supplementary file 5 [file LSA-2022-01690_TableS3.docx]

**Table S3. Sequences of primers or Taqman assay references used for gene expression analysis.**

| **Gene** | **Primer sequences or Taqman assay references** |
| --- | --- |
| *Hprt* | Mm03024075_m1 |
| *Nppa* | Mm01255747_g1 |
| *Nppb* | Mm00435304_g1 |
| *Myh7* | Mm01319006g1 |
| *Myh6* | Mm00440359_m1 |
| *Actc1* | Mm01333821m1 |
| *Tnnt2* | Mm01290256_m1 |
| *C/ebpβ* | Mm00843434_s1 |
| *Cited4* | Mm00550569_s1 |
| *Col1a1* | Mm00801666_g1 |
| *Col3a1* | Mm01254476_m1 |
| *Fn1* | Mm01256744_m1 |
| *Ctgf* | Mm01192933_g1 |
| *Acta2* | Mm00725412_s1 |
| *Tgfb1* | Mm01178820_m1 |
| *Atp2a2* | 5’-CTCCATCTGCTTGTCCAT-3’ (Forward)  5’-GCGGTTACTCCAGTATTG-3’ (Reverse) |
| *Pln* | 5’-TCAGGAGAGCCTCCACTATTGA-3’ (Forward)  5’-TTAAGCTGAGTTGGCATGTTGC-3’ (Reverse) |
